# Diversities of African swine fever virus host-virus dynamics revealed by single-cell profiling

**DOI:** 10.1128/jvi.02035-24

**Published:** 2025-02-11

**Authors:** Xiaoyang Zhao, Yanyan Zhang, Hanying Jia, Lin Lv, Md.Asif Ahsan, Xudong Fu, Rongliang Hu, Zhiqiang Shen, Ning Shen

**Affiliations:** 1 Department of Obstetrics and Gynecology of Sir Run Run Shaw Hospital & Liangzhu Laboratory, Zhejiang University School of Medicine 26441 , Hangzhou, China; 2 Changchun Veterinary Research Institute, Chinese Academy of Agricultural Sciences 595703 , Changchun, China; 3 Liangzhu Laboratory, Zhejiang University School of Medicine 26441 , Hangzhou, China; 4 Shandong Lvdu Bio-Sciences and Technology Co., Ltd., Binzhou, Shandong, China; 5 Shandong Binzhou Academy of Animal Science and Veterinary Medicine, Shandong Academy of Agricultural Sciences 540419 , Binzhou, Shandong, China; Northwestern University Feinberg School of Medicine, Chicago, Illinois, USA

**Keywords:** single-cell RNA sequencing, African swine fever virus, host-virus interaction, macrophage

## Abstract

African swine fever virus (ASFV) causes epidemics with high mortality; however, effective vaccines and therapies remain missing. Here, we depict a temporal single-cell landscape of primary porcine alveolar macrophages (PAMs) exposed to three different virulent ASFV strains *in vitro*. We found that attenuated and low-virulence ASFV strains tend to exhibit higher viral loads than highly virulent strain, which may result from upregulated RNA polymerase subunit genes expression. On the host side, our study highlights the IRF7-mediated positive feedback loop to the activation of the interferon signaling pathway in cells exposed to attenuated and low virulent ASFV strains. Moreover, we unraveled the PAMs populations marked by expressions of the *IFI16* and *CD163*, respectively, which produce high levels of interferon-stimulated genes (ISGs) and IL18 to regulate the host response to different virulent ASFV strains. Collectively, our data provide insights into the complex host-virus interactions with various ASFV strain infections, which may shed light on the development of effective antiviral strategies.

**IMPORTANCE:**

There is still no available research on the temporal transcriptional profile of host cells exposed to different virulent ASFV strains at the single-cell level. Here, we first profiled the temporal viral and host transcriptomes in PAMs exposed to high virulent, attenuated virulent, and low virulent ASFV strains. Our analysis revealed that attenuated and low-virulence ASFV strains tend to exhibit higher viral loads than highly virulent strains, which may result from upregulated RNA polymerase subunit genes expression. We also found a positive feedback loop of the interferon signaling pathway mediated through IRF7 and identified the populations of PAMs marked by *IFI6* and *CD163*, respectively, which produce high levels of ISGs and *IL18* to regulate host response to different virulent ASFV strains. Our study delineated a comprehensive single-cell landscape of host-virus dynamics across ASFV strains with different virulences and would provide an important resource for future research.

## INTRODUCTION

African swine fever is a highly contagious and lethal hemorrhagic disease caused by the African swine fever virus (ASFV). Since its first description in Kenya in 1921, outbreaks of ASFV have swept through Eastern Europe, Africa, and Asia, causing a devastating global threat to pig farming (1). Infected pigs suffer from high fever, internal bleeding, and, most often, death. Currently, there is no effective drug or vaccine available for ASFV (2).

ASFV, the only member of the *Asfarviridae*, is a large, enveloped, double-stranded DNA virus with an average diameter of about 250 nm. Due to its physical size and structural complexity, functional studies of this giant virus are particularly challenging. So far, functions for over half of the ASFV genes remain unknown (1). The virulence of ASFV is reflected by degrees of lethality to the host which is governed by a complex and tightly regulated program of viral gene expression (3). This process involves complex and multifaceted interactions between ASFV genes and host cellular responses, including metabolic pathways, immune pathways, and apoptotic responses (3). However, limited understanding of this process is a main factor that impedes the development of safe and effective ASF vaccines (2).

RNA sequencing has been employed in most previous studies for comprehensive profiling of ASFV gene expression and its interaction with the host transcriptome, but the characterization of individual cells infected with viruses can be masked at the population level (4, 5). In contrast, single-cell RNA sequencing (scRNA-seq) provides a powerful and unbiased approach to overcome this obstacle. scRNA-seq has enabled a greater appreciation of the dynamic changes of virus infection and the complex interactions between viruses and host cells (6). Zheng et al (7) conducted the pioneering scRNA-seq profiling to create a detailed transcriptome profile of ASFV-exposed primary porcine alveolar macrophages (PAMs), offering valuable insights into the intricate interplay between host and viral genes. However, this study focused on a high virulent strain of ASFV, thereby leaving the effective host response to ASFV infection largely unexplored.

Macrophages are the major reservoir for ASFV, and we extracted pig lung lavages that consist of large numbers of leukocytes, with approximately 90% being PAMs (3, 8). We conducted a temporal scRNA-seq analysis of cells from 13 samples containing unexposed PAMs and PAMs that were exposed to three different strains of ASFV, SY18, HuB20, and SY18ΔI226R (referred to as I226R hereafter), at 6, 12, 24, and 48 h post-infection (hpi). SY18 is a high virulent strain that causes nearly 100% mortality in pigs (9, 10). HuB20 is a naturally attenuated ASFV strain that causes 30%–40% mortality in infected pigs, with milder clinical symptoms (11). I226R is a low virulent strain that was derived from the SY18 strain by deleting the *I226R* gene. This deletion resulted in dramatically decreased virulence in which infected pigs show minimal clinical abnormalities (12).

Our analysis characterized the host and virus dynamic transcriptional changes in PAMs exposed to different ASFV strains and revealed that HuB20 and I226R strains were more likely to activate the host interferon-related response through an IRF7-mediated positive feedback loop compared with the SY18 strain. Our results also suggested potential functions of ASFV *I226R* gene in regulating ASFV RNA polymerase and host gene *CEBPB* as key regulator to broad ASFV infection. Overall, our findings may have important implications for understanding and controlling ASFV.

## MATERIALS AND METHODS

### Cell infection and immunofluorescence

All three ASFV strains were obtained from Rongliang Hu Lab as described before (12, 13). All three ASFV strains were passaged in PAMs and stored at −80°C in a biosecurity level 3 laboratory. The PAMs used in this study were prepared from three 45-day-old Landrace piglets. The method for extracting PAMs was described in the previous article (12). Briefly, the intact lung was filled with sterilized phosphate-buffered saline (PBS), and we then collected the bronchoalveolar lavage fluid. Cells were collected via centrifugation at 560 × *g* for 10  min and resuspended using PBS. After removing the erythrocytes and rinsing with PBS, the cell pellets were resuspended, and the cells were grown in RPMI 1640 (Gibco, NY, USA, C11875500) supplemented with 10% fetal bovine serum (Caoyuan Lvye, Huhehaote, China, CTN1002-02). Cells were cultured in an incubator at 37°C under 5% CO2. We performed nucleic acid testing according to China national standards to ensure that there was no contamination with viruses infecting swine, which include ASFV, classical swine fever virus (CSFV), porcine reproductive and respiratory syndrome virus (PRRSV), pseudorabies virus (PRV), porcine parvovirus (PPV), and porcine circovirus 1/2 (PCV1/2).

PAMs infection status was observed by an immunofluorescence assay, with staining with a fluorescein isothiocyanate (FITC)-labeled monoclonal antibody against the ASFV protein p30. Briefly, the PAMs were seeded into a 12-well plate at 80% confluence and exposed to three ASFV strains (multiplicity of infection [MOI] =  3) after seeding. After 06, 12, 24, and 72 hpi, the PAMs were washed once and fixed in 80% cold acetone for 20  min at 4°C. Next, the wells were blow-dried, and mouse anti-p30 serum antibodies (prepared in Rongliang Hu Lab) (12) were diluted 1:50 and 1:200, respectively, in PBS and incubated together for 1 h at 37°C. The goat polyclonal secondary antibody to rabbit IgG-conjugated TRITC (abcam, MA, USA, ab6718) and the goat polyclonal secondary antibody to mouse IgG-conjugated FITC (abcam, MA, USA, ab6785) were diluted 1:500 in PBS and incubated for 1 h together at 37°C in the dark. Each incubation step described above was interspersed by washes three times. We added antifade mounting medium with DAPI (Beyotime, Shanghai, P0131) to the wells. The results were observed under an inverted fluorescence microscope (Olympus, Tokyo, Japan, IX73).

### Sample preparation for scRNA-seq

PAMs in 6-well plates were exposed to ASFV SY18, HuB20, and I226R at 3 MOI, respectively, and PAMs not exposed to ASFV served as the control. After a 1 h incubation period, the viruses were removed, and the cells were washed twice with PBS. Fresh medium was then added to the cells. At the indicated time points (6, 12, 24, and 48 hpi), the cells were gently washed and trypsinized. Single-cell suspensions were washed with PBS containing 0.04% bovine serum albumin (BSA, Beyotime, Shanghai, ST023), resuspended in 200 µL of PBS + 0.04% BSA, passed through a 30 µm filter, and placed on ice. Viable cells were counted based on their exclusion of trypan blue using a LUNA-II Automated Cell Counter (Logos Biosystems, Seoul, South Korea). The appropriate number of cells to achieve a targeted cell input of 10,000 cells per condition was used to generate the gel bead-in emulsion (GEMs). The Chromium Next GEM Single-Cell 3' GEM, Library & Gel Bead Kit SingleR.1 (10× Genomics, Pleasanton, CA, USA) was used for GEM generation, cDNA synthesis, and library preparation following the manufacturer’s instructions. The libraries were sequenced using a NovaSeq 6000 sequencer (Illumina, San Diego, CA, USA). To mitigate the potential impact of technology batch effects, we ensured that all samples are processed by the same researcher and using the same reagents and that the library construction and sequencing procedures are carried out simultaneously.

### RNA isolation and real-time quantitative PCR (qRT-PCR)

PAMs cultured in 6-well plates exposed to three ASFV strains were collected at 6-, 12-, 24-, and 48 h post-inoculation (hpi), and PAMs not exposed to ASFV were collected at 0 hpi. Total RNA was isolated using the Total RNA Kit I (Omega Bio-Tek, GA, USA) and reverse transcribed with HiScript II Q RT SuperMix (Vazyme, Nanjing, China, R223-01) following the manufacturer’s instructions. qRT-PCR was performed using ChamQ Universal SYBR qRT-PCR Master Mix (Vazyme, Nanjing, China, Q712-02/03) on a Real-Time PCR System (Roche, Basel, Switzerland, LightCycler 480). The relative mRNA expression was calculated using the comparative cycle threshold (2^-ΔΔCt^) method and normalized to porcine β-actin mRNA levels. The results were analyzed using GraphPad Prism 10 software (Version 10, La Jolla, CA, USA). The primer sets (Table 1) were used to detect the genes.

**TABLE 1:** Primers used for determination of gene expression by qPCR

Primer	Forward (5' to 3')	Reverse (5' to 3')
*IRF3*	AAGAGGCTGGTGATGGTCAAG	TGGCTGTTGGAAATGTGCAG
*IRF7*	AAAACCAACTTCCGCTGTGC	AGCTCGGGGTTGATCATATACAC
*IFIT1*	AATTGGCAGCCTAAAGGAGAGG	TGTGGGCTTTTGTTGCACAG
*IL18*	GACCTGGAATCGGATTACTT	AACAGTCAGAATCAGGCATA
*IFI6*	CGGTGGAGAGGAGACAGACA	TCGAGTTGCTTGCTGACAGT
*CD163*	CCCATGTGGTGTGCAAACAG	TCCATGTCTCCTCTGAGGGG
*SIGLEC1*	CCCTGGACTTCCATGCCAAT	TTGGGTGCTTTGGTGACCTT
*ACTIN*	GGACTTCGAGCAGGAGATGG	AGGAAGGAGGGCTGGAAGAG
*H359L*	GATCGAGGATTCCACGGACC	AAAACAGCACAAGTTCCGGC
*C147L*	TCATGGATGACCTCGTGGAG	TAACAGCGGGATGCCCATTT
*D205R*	TCGTAAGATGGTGCTGTTGGA	TTTGCACAAAGTCTCCCGGT
*CP80R*	ACCTGTGGTGTGTTTTACCTGT	AAATGAGTGCGACAACACACC
*EP1242L*	AGCGCCCCATTGTAAACAGA	CCTACCGACGTCCAACCAAA
*NP1450*	TTTCCTTTGCACGAACGCTG	CAAAAAGCTTCCCACCGTGG
*I226R*	TTCCTTTTAGCGGTCAAGCG	GGTTTCGGAGGTTACCAATACC
*P72*	AGTTATGGGAAACCCGACCC	CCCTGAATCGGAGCATCCT
*CP80R*	CAAAATGGGCGGAACATTGC	AATGAGTGCGACAACACACC

### Processing scRNA-seq data

The procession of 10× gene expression data were performed using 10x Genomics CellRanger software (version: 3.0.2, 10x Genomics, Pleasanton, CA, USA) (14). Sequence data were mapped into a manually combined reference genome of Ensemble Sus scrofa genome (Sus_scrofa.Sscrofa11.1.dna_sm. toplevel.fa) and ASFV genome (SY18: GenBank accession no. MH766894; HuB20: GenBank accession no. MW521382; I226R: see the supplemental text). The genome difference between SY18, HuB20, and I226R can be seen in Table S3. Unique molecular identifiers (UMIs) were quantified based on the manually combined genome paired annotation files. With the UMI count gene-by-cell matrix, we analyzed the data using the *Seurat* R package (v 4.3.0) (15). Only high-quality cells that matched the following three criteria were retained for the downstream analysis: (i) mitochondrial RNA genome <10%; (iii) number of detected host genes greater than 500 but less than 7,500; and (iii) the number of total host gene UMIs between 1,000 and 60,000. We detected and excluded doublets using Doubletfinder (v3) (16) with default parameters. From the filtered cells, the UMI of host gene expression levels were normalized by converting them to relative values by the total UMI per cell and multiplying this by 10,000, and then, the log-transformed the result. Viral gene expression levels were normalized with counts per million mapped reads (CPM) as the UMI number of a given viral gene in a given cell divided by the total viral UMI number of a given cell and multiplied by 1,000,000, then transformed into log2(CPM + 1). Notably, when the normalized host/viral genes were calculated, only the UMI number of host/viral genes was considered. The FindVariableFeatures function was used to obtain the top 2,000 highly variable host genes (HVGs) of each sample. We merged scRNA-seq data of unexposed and exposed cells. The merged data set on all cells was then used to scale and center the host genes and compute the principal components (PCs). After principal components analysis (PCA) to reduce dimensionality and build k-nearest neighbor graphs (k = 20) of the cells with the function *FindNeighbors* based on the Euclidean distance in the 30-dimensional PC space. For classifying filtered cells, the clustering parameter resolution was set to be 0.3 with the function FindClusters in Seurat. Next, the function RunUMAP with dimensions parameters (1:30) was used to reduce high dimensions into two dimensions (2D) for visualization.

### Calculating normalized viral load and proportional viral gene expression

We normalized each viral gene expression in each cell as mentioned above and sum all the normalized viral gene expression in each cell which we defined as normalized viral load. For calculating the proportional viral gene expression in sample S, we defined E_g,c_ as the normalized expression of viral gene g (g = 1, 2, …, G) in cell c (c = 1, 2, …, C). Therefore, the proportional viral gene expression was calculated as follows: $Proportions_{C\epsilon S}(c,g)=\frac{\sum_{c=1}^{c}E_{g,c}}{\sum_{g=1}^{G}\sum_{c=1}^{C}E_{g,c}}$

### Identifying cell types

We employed SingleR (v1.6.1) (17) for unsupervised identification of specific cell types. Marker genes corresponding to these cell types were retrieved from CellMarker (18). Cell type annotations were manually adjusted based on the expression patterns of cell type-specific marker genes in each cell. This ensured that the final cell type assignments aligned with the expression patterns of their specific marker genes (Table S3).

### Identifying differential expressed genes (DEGs)

DEGs between cells from different clusters, exposed to different ASFV strains and post-infection time (hip), were identified by using FindMarkers or FindAllMarkers function in R package Seurat. The genes were selected as DEGs only if the average difference was greater than 0.5, the percentage of expressed cells in the corresponding group was greater than 25%, and FDR was less than 0.05. Gene ontology (GO) analysis was performed on the DEGs by using the clusterProfiler (v4.0.5) (19) R package.

### Collecting different gene sets and calculating gene scores

The gene sets used in this study were collected from the following sources. ISGs and inflammation-related genes markers were collected from a previous study (7) (Table S3). For each single cell, gene scores were calculated by using AddModuleScore function in R package Seurat. Briefly, the AddModuleScore function calculates the average expression levels of each gene set on a single-cell level, subtracted by the aggregated expression of control feature sets. The calculation of ISG score and inflammation score was performed as follows: ISGs and inflammation-related genes were collected from a previous study (7) (Table S3). For each cell, we computed the average expression levels of ISGs or inflammation-related genes and subtracted the aggregated expression of control feature sets (randomly selected). The metabolic scores for each sample were calculated by using the package scMetabolism (20).

### Constructing transcriptional regulator networks

Based on the raw UMI count data, transcriptional regulator networks were established using the R package SCENIC (21). DEGs between different ASFV strains exposed cells were selected at all time points for this analysis. Since no regulator resources for the pig, the human regulator database was used. The DEGs motifs around the 10 kb transcription start site (TSS) were analyzed for transcription factor (TF) enrichment. Gene correlation analysis was performed first, and log2-transformed UMI count data were used to infer the regulator network. Only highly confident annotations and connections with high weights (CoexEeight ≥0.03) were maintained, and regulators with more than three connections were considered.

### Identifying host genes expression related with viral loads

Differential expression tests analysis was performed between the expression level of each host gene and viral loads by using MAST (22). We used the normalized viral load as a continuous exogenous variable and only considered cells with normalized viral load≧1. Differential expression *P*-values were corrected for multiple hypothesis testing using the Benjamini method.

### Single-cell pseudotime trajectory reconstruction and analysis

Single-cell pseudotime trajectories were constructed using Monocle (v2.20.0) (23). Briefly, we first selected a set of ordering genes that showed differential expression between the cells exposed to different ASFV strains. Then, Monocle uses reversed graph embedding, a machine learning technique to generate a parsimonious principal graph, and reduces the given high-dimensional expression profiles to a low-dimensional space. Single cells were projected onto this space and ordered into a trajectory with branching points. Subsequently, the cells were ordered according to their progression through the developmental program. Monocle measures this progress as pseudotime. In this study, single-cell trajectory analysis of cells exposed to different ASFV strains was performed as needed.

### Enzyme-linked immunosorbent assay (ELISA)

ELISA kits were used to measure the porcine protein levels of CYP2E1 (Wuhan ColorfulGene, China, cat no. JYM0361po) and IL18 (Jiubang Biotechnology, China, cat no. QZ-21108). All operations were conducted in strict accordance with the instructions provided by the kit. Briefly, PAMs exposed to different ASFV strains at 48 hpi (10 µL) and diluent solutions (40 µL) were added to the wells on a 96-well plate separately. Next, each well was incubated with HRP-labeled secondary antibodies at 37°C for 60 min. Finally, we measured the optical density of each well at 450 nm after adding 50 µL termination solution within 15 min.

### Western blotting

Cell lysates were subjected to 10% SDS-PAGE and transferred to nitrocellulose membranes. The membranes were blocked for 1.5 h at room temperature in Tris-buffered saline containing 10% nonfat dry milk and 0.05% Tween 20 (1× TBST), and further incubated at 4°C for 12 hours with the CEBPB antibodies (proteintech, China, cat no. 23431-1-AP). The membranes were washed with 1× TBST, incubated with horseradish peroxidase (HRP)-labeled goat anti-rabbit IgG or anti-rabbit IgG antibody (Beyotime, China) at room temperature for 45 min, and treated with enhanced chemiluminescence (ECL) reagent (Thermo Fisher Scientific, USA).

### Viral replication kinetics

To evaluate viral replication of SY18, HuB20 and I226R strain *in vitro*, PAMs in 6-well plates were exposed to ASFV SY18, HuB20, and I226R at 3 MOI. Next, the culture was collected at 6, 12, 24, and 48 hpi, and freeze-thawing was repeated three times. The TCID_50_ of SY18, HuB20, and I226R at different time points were assayed by lysis of the liquid-nitrogen freeze-thawed virus cell culture. Virus titration was performed on PAMs in 96-well plates observed by an immunoﬂuorescence assay, with staining with FITC-labeled monoclonal antibody against the ASFV protein p30. Brieﬂy, virus suspensions were 10-fold diluted and inoculated onto the cell monolayer with 80% confluence. After cultivation for 5 days at 37°C, PAMs were ﬁxed with 80% pre-cooled acetone and stained with the FITC-p30 antibody (prepared in our laboratory) for 1 h at 37°C. After washing with cold PBS three times, the samples were observed with a ﬂuorescence microscope. The Reed-Muench method was used to calculate the virus titer.

### Sample distribution of cell populations

We calculated the *Ro/e* for each cell population in different samples to quantify the sample preference of each cell population (24). The expected cell numbers for each combination of cell population and samples were obtained from the chi-squared test. One cell population was identified as being enriched in a specific sample if *Ro/e* >  1.

### Statistical analysis

**Fig 1:**
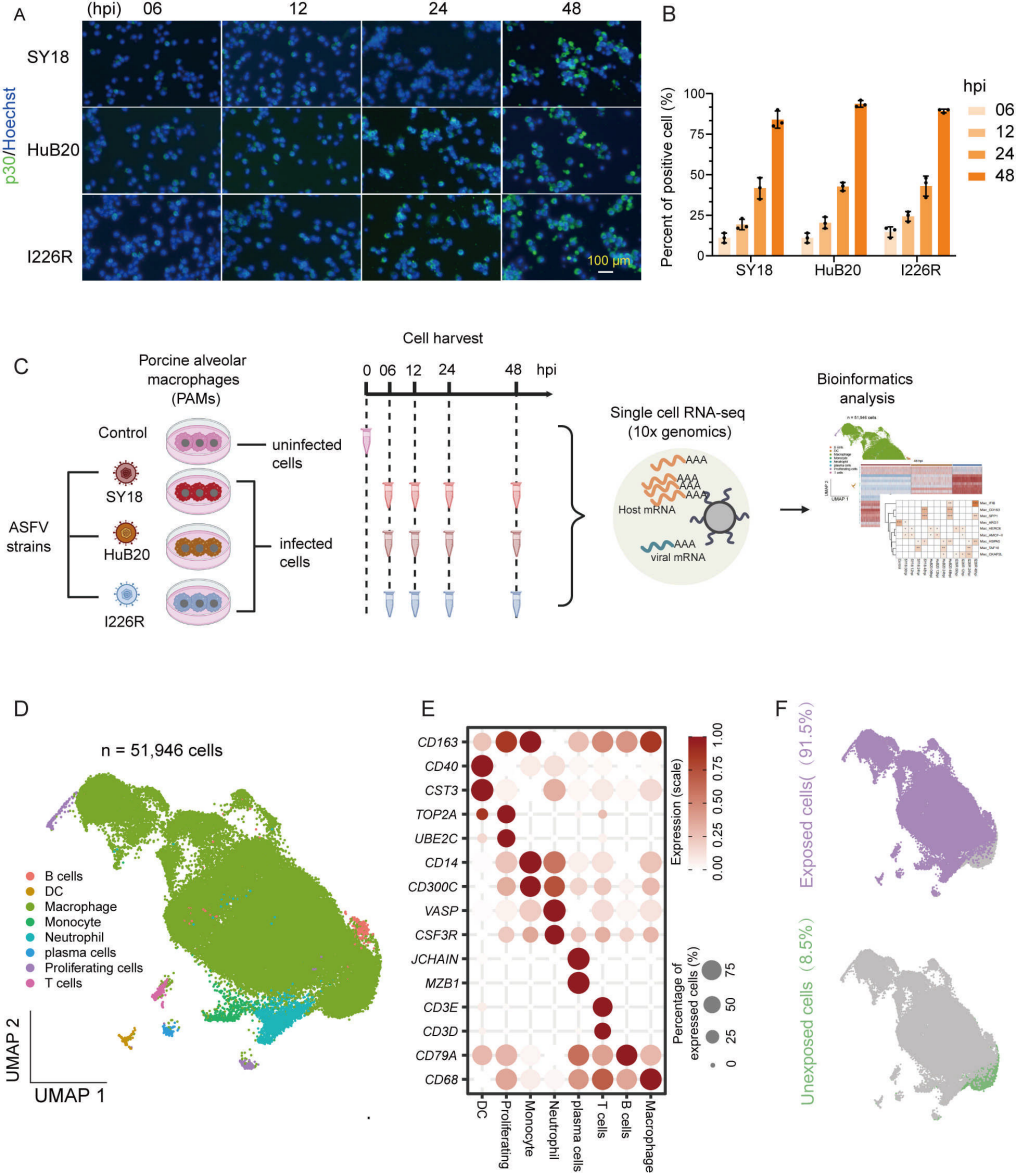
Landscape of single-cell transcriptome dynamics in PAMs exposed to ASFV. (**A**) Immunofluorescence of ASFV viral protein p30 in cells exposed to different ASFV strains at different hpi. Scale bar = 100 µm. (**B**) Bar plots showing the proportion of p30 positive cells for each ASFV strain at the different hpi. Data are shown as mean value ± SD, *n* = 3. (**C**) Schema of this study design. (**D**) UMAP plots showing the cell types in the integrated single-cell transcriptomes of 51,946 cells derived from ASFV unexposed and exposed cells. (**E**) Dot plot showing scaled expression levels of cell type–specific genes in unexposed and exposed cells. Expression levels in each gene are scaled, ranging from 0 to 1. The dot color and size indicate expression levels and the percentage of expressed cells, respectively. DC, dendritic cell. (**F**) UMAP plots showing the distribution of unexposed cells and exposed cells.

Single-cell RNA-seq data were carried out on 13 samples as mentioned in Fig. 1, and a total of 51,946 individual cells were used in the downstream analysis for this study.

**Fig 2:**
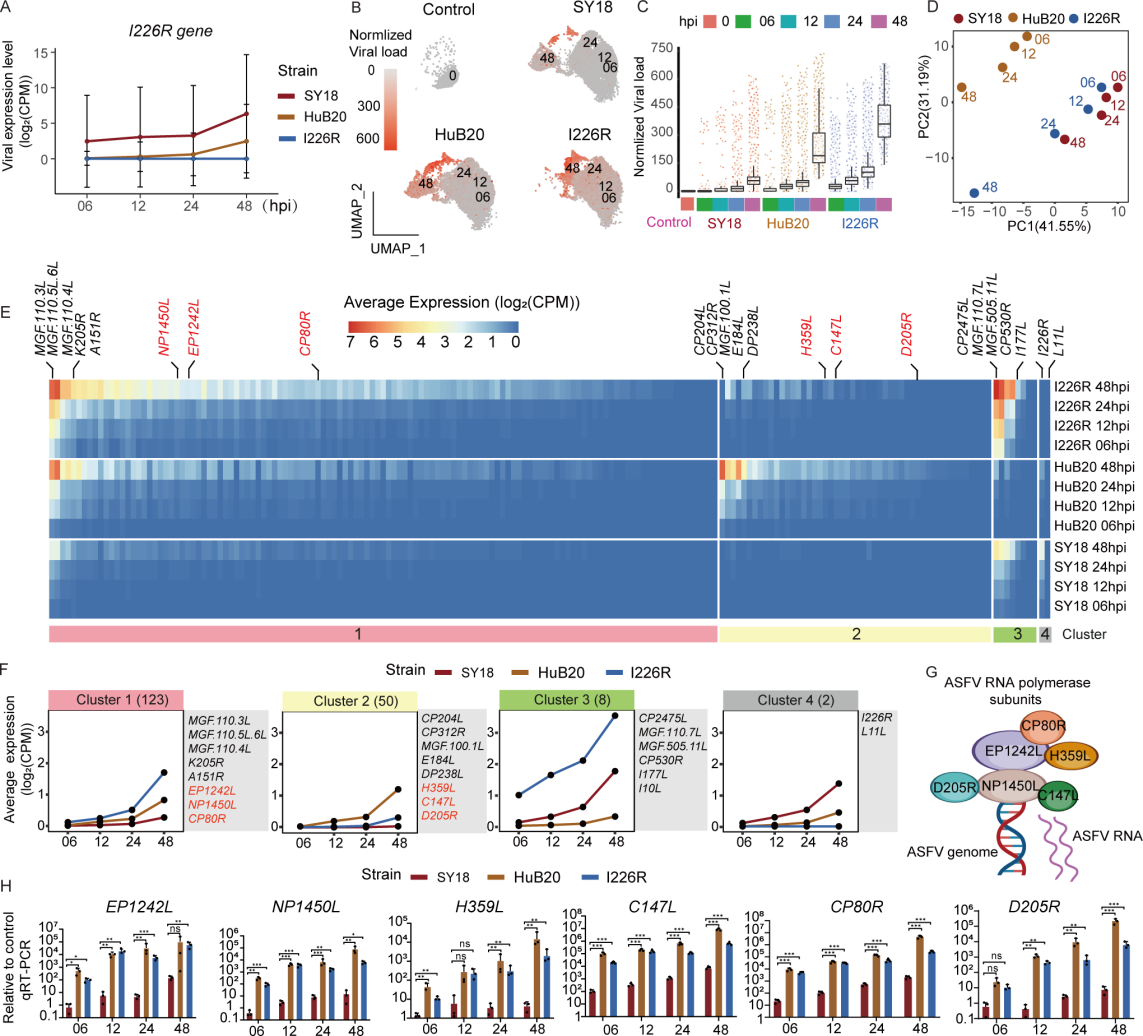
Attenuated and low-virulence ASFV strains tend to exhibit higher viral load compared with highly virulent ASFV strain. (**A**) Line plots showing the expression levels of representative viral genes. Gene expression levels are quantified with counts per million mapped reads (CPM). Data are shown as mean value ± SEM. (**B**) UMAP plot showing the normalized viral load in cells in each virus strain. 0, 06, 12, 24, and 48 mean hours post-infection (hpi). (**C**) Box and point plot showing the viral load in each sample. Data are shown as mean ± SEM. (**D**) PCA plot showing the distribution of each sample by the proportional viral gene expression. (**E**) Heatmap plot showing expression levels of ASFV genes in each sample. (**F**) Line plots showing average expression levels of viral genes in four gene clusters. The number of viral genes in each gene cluster is indicated in parentheses. (**G**) Brief schematic overview of ASFV RNA polymerase to induce ASFV transcription. (**H**) Bar plots showing qRT-PCR results of ASFV RNA polymerase subunits genes transcription. ns, not significant; **P < 0.01 and ***P < 0.001 (two-tailed t test). Data are shown as mean value ±SD, *n* = 3.

The experimental data (Fig. 2H, Fig. 4E, Fig. 5G, and Fig. 6E; Fig. S2A; Fig. S5B, D, and F) were statistically analyzed using a two-tailed *t*-test with GraphPad 10 software. Statistical significance was defined as *P* < 0.05. One, two, and three asterisks indicated *P < 0.05, **P < 0.01, and ***P < 0.001, respectively; ns indicated not significant.

The bioinformatics data analyses were statistically analyzed using R (4.3.0). Correlation and corresponding statistical significance were calculated based on Spearman’s rank correlation coefficient. The differences in categorical variables between the two groups were analyzed using two-tailed *t*-test. Functional enrichment was assessed by a hypergeometric test, which was used to identify a priori-defined gene sets.

*P* values were further adjusted by Benjamini-Hochberg correction, and adjusted *P* values less than 0.05 were considered statistically significant.

## RESULTS

### Landscape of single-cell transcriptome dynamics in PAMs exposed to ASFV

To investigate the host-virus dynamic transcriptional changes during ASFV infection at single-cell resolution, we exposed PAMs to three ASFV strains: SY18, HuB20, and I226R, representing high, attenuated, and low virulent strains, respectively. We collected phosphate-buffered saline (PBS)-treated PAMs as the control and PAMs exposed with three ASFV strains at 6, 12, 24, and 48 hpi. The expression of p30, which is encoded by the viral gene *CP204L*, was detected in the ASFV-exposed groups by immunofluorescence assay. The percentage of p30-positive cells increased gradually over time with similar kinetics in the three different ASFV strains, indicating productive infection of PAMs to all three strains of ASFV (Fig. 1A and B). We then utilized 10× genomics technology to profile the transcriptomes of PAMs from all samples (Fig. 1C). In this study, cells from the PBS control group were designated as un exposed cells, whereas cells from the samples exposed to ASFV were categorized as exposed cells. After applying standard quality control, we obtained a total of 4,418 high-quality unexposed cells and 47,528 exposed cells (the number of high-quality cells exposed to each virus strain can be seen in Fig. S1A). An average of 3,886 genes and 21,431 UMI were found in each sample after filtration ( Fig. S1B). We visualized these data using uniform manifold approximation and projection (UMAP) for nonlinear dimensionality reduction analysis. By performing supervised clustering analysis of the transcriptomes and utilizing well-known marker genes, we identified eight distinct cell types (Fig. 1D and E), among them macrophages were the predominant cell type (93.8%), which aligns with findings from a prior study (7). Since macrophages are the main target cells of ASFV infection, we focused on macrophages in the subsequent analyses. In addition, the UMAP plot demonstrated a clear difference between unexposed and exposed cells, suggesting that ASFV infection induced global transcriptomic changes in the host cell (Fig. 1F; Fig. S1C). According to previous studies, exposed cells could be further separated into bystander cells and infected cells (7, 25). The infected cells and bystander cells in each sample were defined by the Otsu’s thresholding after logarithmic transformation (Fig. S1D) (25).

### Attenuated and low-virulence ASFV strains tend to exhibit higher viral load compared with highly virulent ASFV strains

By aligning reads to the genomes of the three ASFV strains, we were able to analyze the viral transcripts in each PAM cell. We first confirmed that the *I226R* gene is not expressed in the I226R strain, although being expressed in both the SY18 and HuB20 strains, confirming the successful knockout of the *I226R* gene in our I226R strain (Fig. 2A).

A total of 31,803 (72.6%) exposed cells were detectable at least one UMI for viral transcripts. Notably, viral load consistently increased across all ASFV strains as the infection progressed (Fig. 2B and C). Interestingly, the attenuated and low-virulent ASFV strains, HuB20 and I226R, exhibited a higher viral load and infected cell rate compared with the highly virulent strain SY18 (Fig. 2B and C; Fig. S2A). To validate these findings, we performed quantitative real-time PCR (qRT-PCR) to measure the mRNA levels of the *p72* gene, which is widely used as an indicator of ASFV mRNA abundance (26). Consistently, the attenuated and low-virulent ASFV strains, HuB20 and I226R, exhibited higher *p72* levels compared with the virulent strain SY18 (Fig. S2B). Remarkably, from 6 to 48 hpi, although our data demonstrate higher transcriptional abundance (viral load) in I226R compared with SY18, our viral titer experiments indicated a slightly lower viral titer for I226R, aligning with our previous research (Fig. S2C). This finding indicates that despite elevated transcript levels, the viral transcripts from I226R may not be efficiently translated or assembled into new virions. Furthermore, we observed a gradual decline in the diversity of host genes as the viral load increased in the exposed cells, suggesting a potential suppression of host gene diversity caused by ASFV replication. (Fig. S2D).

We next visualized the proportional viral gene expression at 48 hpi among the three ASFV strains (Fig. S2E). Surprisingly, despite I226R having a higher viral load than SY18, SY18 and I226R exhibited similar highly proportionally expressed viral genes such as *CP2475L*, *CP530R*, and *MGF.505.11L*, implying that SY18 and I226R share similar viral gene expression patterns. We then performed PCA analysis on all samples based on the proportional viral gene expression. The expression pattern of viral genes in I226R-exposed cells was similar to that in SY18-exposed cells, whereas viral genes in HuB20-exposed cells exhibited a unique expression pattern (Fig. 2D). More importantly, the gene expression pattern of the I226R transcript was faster than that of SY18 at each time point. For example, the viral gene expression pattern of I226R at 6 hpi exhibited greater similarity to SY18 at 12 hpi. Likewise, the gene expression profile of I226R at 12 hpi exhibited a closer resemblance to that of SY18 at 24 hpi (Fig. 2C). This phenomenon can also be found in infected cells (Fig. S2F). This result indicates that the I226R strain exhibits a faster transcription rate compared with the SY18 strain.

To further examine the differences in viral gene expression among various strains of ASFV, we derived four clusters based on the average virus gene expression across time points (6, 12, 24, and 48 hpi) (Fig. 2E and F; Table S1). Notably, the expression levels of viral genes in clusters 1 and 2 were higher in cells exposed to I226R and HuB20. In particular, we noticed that viral genes encoding ASFV RNA polymerase subunits (highlighted in red in clusters 1 and 2) showed increased expression in I226R and HuB20 exposed cells (Fig. S2G). These findings were further corroborated by independent qRT-PCR experiments (Fig. 2H). Considering that ASFV relies on its own RNA polymerase for transcription (Fig. 2G) (27, 28), this observation may explain the higher viral load in HuB20 and I226R, as well as the accelerated transcription rate observed in the I226R strain compared to the SY18 strain. *I226R* was also described as an important virulence-related gene, evidenced by ectopic expression of I226R significantly inhibited the host innate immune response (12). We noticed that *I226R*, along with the transmembrane viral gene *L11L* in cluster 4, showed upregulated expression in SY18 exposed cells, implying their potential role in regulating ASFV virulence. In addition, viral genes in cluster 2, such as *CP204L*, *CP312R*, *MGF100.1L*, and *E184L* showed increased expression in HuB20 exposed cells, whereas hardly any viral transcripts were detected in SY18 exposed cells (Fig. 2E and F). Notably, CP204L is a multifunctional protein involved in various stages of the ASFV life cycle (29). CP312R and E184L have recently been linked to ASFV immune evasion (30). However, functional annotation on other viral genes in cluster 2 is currently lacking, which warranted further investigation.

In cluster 3, *CP2475L*, *MGF. 110.7L*, *MGF. 505.11L*, *I177L*, and *I10L* were highly expressed in the I226R strain, followed by the SY18 and HuB20 strains. Interestingly, *I177L* is a ASFV virulence-related gene, and knockout of *I177L* can significantly reduce the virulence of ASFV *in vivo* (31, 32). In our data, the expression of *I177L* in I226R strain was higher than that in SY18, indicating that the expression level of *I177L* may be compensatory after *I226R* knockout. Considering that *I226R* and *I177L* are both ASFV virulence genes and can significantly reduce the virulence of ASFV after knockout, this result suggests that *I226R* and *I177L* may have a synergistic effect.

In summary, these results demonstrated that attenuated and low virulent ASFV strains exhibit higher viral loads compared wth the high virulent ASFV strains, potentially due to the upregulated expression of ASFV RNA polymerase subunit genes. This analysis provides insights into the intracellular dynamics of viral transcription of ASFV strains with varying levels of virulence.

### Inflammatory response and metabolism activation of PAMs were enhanced after exposed to ASFV

**Fig 3:**
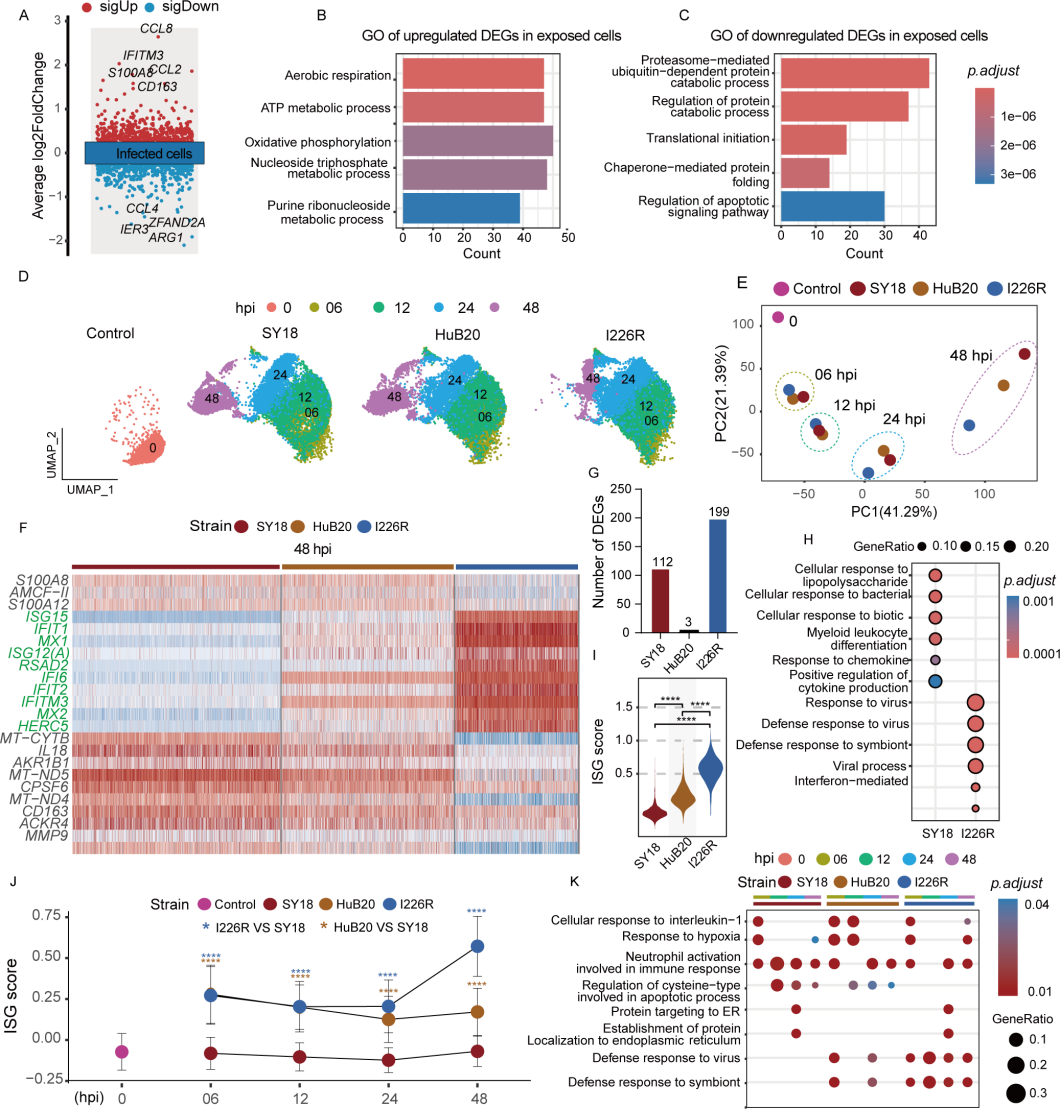
The interferon pathways were activated in PAMs exposed to attenuated and low virulent ASFV. (**A**) Dot plot showing the DEGs between unexposed and exposed cells. Each dot corresponds to a gene (red means upregulated and blue means downregulated). (**B**) Bar plots showing enriched GO terms of upregulated genes in exposed cells. (**C**) Bar plots showing GO terms of downregulated genes in exposed cells. (**D**) UMAP plot showing the distinct composition of cells without or with exposed to three ASFV strains (exposed to SY18, HuB20, and I226R). The different colors indicated different hpi. (**E**) PCA plot showing the distribution of each sample by the host gene expression. (**F**) Heatmap depicting the expression levels of top 10 genes by log2FC enriched in each ASFV strains exposed PAMs at 48hpi. ISG genes are highlighted using green color. (**G**) Bar plots showing the number of DEGs among three ASFV strains exposed PAMs at 48 hpi. (**H**) Dot plots showing enriched GO terms of genes highly expressed in SY18 or I226R exposed cells at 48 hpi. (**I**) Violin plots showing ISG score among three ASFV strains exposed PAMs at 48 hpi. *P < 0.05, **P < 0.01, and ***P < 0.001 (two-tailed t test). (**J**) Line plot showing ISG score for the cells exposed by three ASFV strains at each hpi. The lines indicate the median trend of the ISG score. *P < 0.05, **P < 0.01, and ***P < 0.001 (two-tailed t test). (**K**) Dot plots showing GO terms of genes highly expressed in cells exposed to three ASFV strains at each hpi.

We have demonstrated global transcriptomic alterations in host PAMs following ASFV infection (Fig. 1F). Interestingly, we observed that the gene expression profiles of exposed cells and infected cells are very similar (Fig. S3A). To further investigate the characteristics of the PAMs transcriptome under ASFV exposure, we identified DEGs between unexposed and exposed cells. For the exposed cells, the expression levels of *CD163*, *IFITM3*, and *S100A8* were higher compared with those in the unexposed cells (Fig. 3A). Among them, CD163 has been recognized as one of the potential cofactors for ASFV to invade cells (33). Thus, we reasoned that CD163 over-expression might increase host susceptibility to ASFV infection. Other upregulated genes, such as *S100A8*, *CCL8,* and *CCL2,* have been reported to enhance macrophage responses to pro-inflammatory stimuli (34). On the contrary, the anti-inflammatory gene IER3 and *ARG1* (35, 36) were downregulated in exposed cells (Fig. 3A). Taken together, these results suggested that ASFV-exposed cells likely shift PAMs towards a more pro-inflammatory phenotype.

We next performed Gene Ontology (GO) analysis for these DEGs and found that genes highly expressed in ASFV-exposed cells were significantly enriched in energy metabolism-related pathways (Fig. 3B), whereas the downregulated genes were enriched in the protein modification process and translational initiation pathway (Fig. 3C). Previous reports have shown that ASFV could seize organelles to synthesize a large number of metabolites required for self-replication (37). To obtain a more comprehensive assessment of the metabolism pathways involved, we employed the *scMetabolism* pipeline to quantify metabolic activity at the single-cell resolution (20). The results showed that most of the metabolic pathways were activated after ASFV infection (Fig. S3B), such as the tricarboxylic acid cycle (TCA) and glutathione metabolism pathway (indicated with red). ASFV infection has been proven to induce the increased activity of the TCA cycle (37), and oxidized glutathione has been found to induce disassembly of the ASFV capsid (38). The remaining metabolic pathways that have not been reported need to be studied in the future.

### The interferon-related pathways were activated in in PAMs exposed to attenuated and low virulent ASFV

After investigating the global patterns of gene expression between unexposed and exposed cells, we then sought to figure out the molecular traits exhibited by cells exposed with different virulent ASFV strains. To achieve this, we employed UMAP (Fig. 3D) and PCA (Fig. 3E) analysis on host cells exposed by different ASFV strains. The disparities of the host gene expression pattern between the three ASFV strains became much more evident as the infection time increased. Considering the nearly identical viral genome between the SY18 and I226R strains, we wondered whether the host gene expression difference reflects disparate cell fate or a continuous temporal response. Thus, we conducted a pseudo-time trajectory analysis to infer lineage relationships among cells exposed to three ASFV strains. The results revealed that most SY18-exposed cells were found in the upper half of the trajectory diagram, whereas I226R-exposed cells were primarily located in the lower half of the trajectory diagram (Fig. S3C). Cells exposed to SY18 and I226R were not distributed on the same trajectory, implying that the discrepancy of exposed host cell transcriptomes between SY18 and I226R was an indication of different cell fate rather than temporal transitions during infection.

Since PAMs at 48 hpi exhibited the most distinct disparities in host gene expression among various ASFV infections, we then focused on examining the DEGs in PAMs exposed to different ASFV strains at 48 hpi. The heatmap of these DEGs revealed that host cells exposed to HuB20 and I226R exhibited high expression levels of interferon-stimulated genes (ISGs, indicated in green), including *ISG15*, *MX1*, *IFI6*, and *IFITM3*, compared with SY18-exposed cells (Fig. 3F). We then summarized the number of DEGs and found that they were predominantly concentrated in cells exposed to the high virulent ASFV strain SY18 and the low virulent ASFV strain I226R, whereas only a few DEGs were found in cells exposed to the attenuated ASFV strain HuB20 (Fig. 3G). This result indicates that HuB20-exposed cells are in a state between SY18- and I226R-exposed cells. This result aligned with the distinct ASFV pathogenicity between SY18 and I226R. GO analysis results showed that genes involved in anti-viral response and interferon-mediated pathways were commonly upregulated in I226Rexposed cells. In contrast, genes upregulated in SY18-exposed cells were related to biotic stimulus (Fig. 3H). Previous studies have shown that hosts infected with naturally attenuated ASFV strains or ASFV strains with deletions in various genes can induce a potent antiviral immune response. This response particularly targets the interferon pathway, thereby triggering robust immunity against ASFV infection (39–44). However, virulent ASFV strains can evade the immune response by inhibiting the activation of the interferon pathway in host cells through the encoding of a variety of interferon antagonist proteins (4, 12, 45, 46).

In order to systematically evaluate the type I interferon activity, we calculated the ISG score based on a curated list of interferon-related genes (7) (Table S3). We observed I226R and HuB20-exposed cells exhibited similar and higher ISG scores than SY18-exposed cells at 48 hpi, suggesting that the intensity of interferon pathway activation was higher in attenuated and low virulent ASFV strains compared with high virulent ASFV strain (Fig. 3I).

To characterize the level of interferon pathway activation during the whole infection process, we also evaluated ISG score in each ASFV strain across infection time points (Fig. 3J). The ISG scores for HuB20 and I226R were significantly higher than that of SY18, from 6 hpi to 48 hpi in both exposed cells and infected cells (Fig. 3J; Fig. S3D). This result implies that from the early phase of infection, attenuated and low virulent ASFV strains could stimulate the interferon pathways to promote ISG expression. In contrast, the ISG score for SY18 was consistently similar to the control group at each time point after infection, implying that the interferon pathway activation was consistently suppressed in SY18-exposed cells (Fig. 3J). Moreover, in the pseudo-time trajectory analysis, we observed that as the infection duration increased and the viral load rose, the ISG score in cells exposed to SY18 did not show a significant increase (upper half of the trajectory diagram) (Fig. S3E). In contrast, the ISG score in cells exposed to HuB20 and I226R increased markedly (bottom of the trajectory diagram). These findings were further supported by GO analysis that defense response to virus pathway was significantly enriched in cells exposed to HuB20- and I226R-infected cells, but not in cells exposed to SY18 (Fig. 3K). Intriguingly, our analysis revealed a distinct pattern regarding cells exposed to HuB20. Although the ISG score was relatively high at 6 hpi, it gradually declined from 6 to 48 hpi, in contrast to the significant increase observed in cells exposed to the I226R strain (Fig. 3J). Additionally, we identified a negative correlation between viral load and ISG score in cells exposed to and infected by HuB20 (Fig. 3F and G). These findings suggest that upon exposure to the HuB20 strain, unlike SY18 which immediately suppresses cellular immunity, there is an initial activation of the cellular immune response. However, as the virus replicates within the cell, the immune response appears to gradually diminish, potentially due to an inhibitory effect exerted by the replicating virus.

Collectively, these data suggest that interferon pathway activation might be a key player determining the virulence of ASFV. For strains with different virulence levels, the inhibition of cellular immunity occurs at distinct stages of infection.

### The interferon pathways were activated through the IRF7-mediated positive feedback loop in PAMs exposed to attenuated and low-virulent ASFV

**Fig 4:**
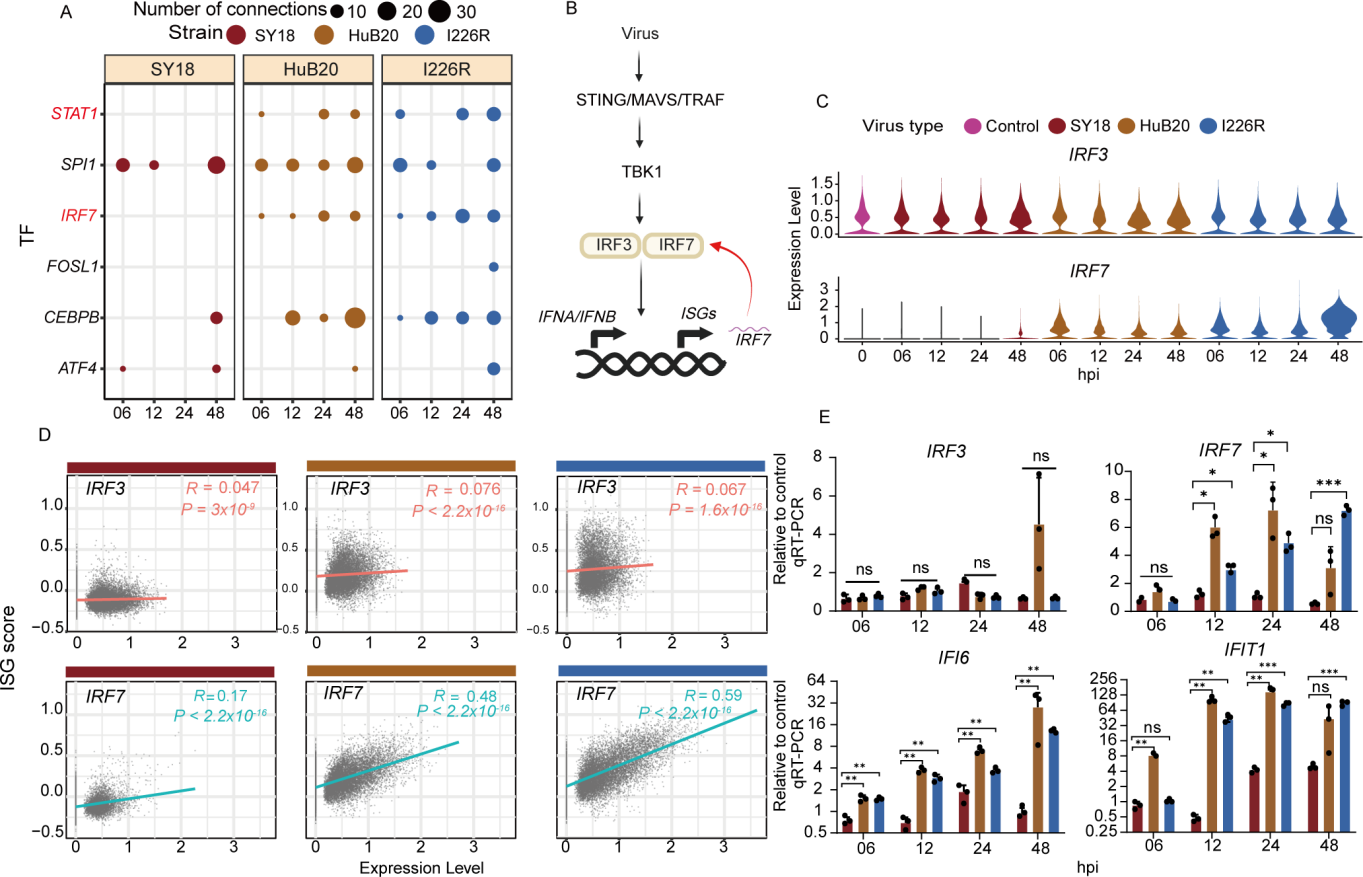
The interferon pathways were activated through the IRF7-mediated positive feedback loop in PAMs exposed to attenuated and low virulent ASFV. (**A**) Dot plots showing potential key regulators in cells exposed to three ASFV strains. The dot color and size indicate ASFV strains and the number of connections. (**B**) Brief schematic overview of virus induced IFN activation pathway. (**C**) The violin plots show the expression levels of *IRF3* and *IRF7* among three ASFV strains exposed PAMs. (**D**) Scatter plots showing the correlation of normalized expression levels of *IRF3* and *IRF7* (horizontal axis) with ISG score (vertical axis) in cells exposed to three ASFV strains. (**E**) Bar plots showing the qRT-PCR results of the relative transcriptional level of *IRF3, IRF7, IFI6,* and *IFIT1* in cells exposed to different ASFV strains. ns, not significant; *P < 0.05, **P < 0.01 and ***P < 0.001 (two-tailed t test). Data are shown as mean value ± SD, *n* = 3.

To figure out the potential core regulators among the cells exposed to the three ASFV strains, we further conducted single-cell regulatory network inference and clustering (*SCENIC*). Our analysis revealed that two transcription factors (TF), *STAT1* and *IRF7,* were significantly enriched in host cells exposed to HuB20 and I226R, but not in cells exposed to SY18 (Fig. 4A; Table S2). Remarkably, a multitude of downstream gene targets associated with these TFs were implicated in the ISGs family (Fig. S4A), signifying their potential involvement in regulating ASFV-induced interferon production. *STAT1* was a well-characterized key regulator of IFN-γ and cytokine signaling in virus infection (47, 48). As for *IRF7*, previous studies have suggested its collaboration with *IRF3* in orchestrating the regulation of type I interferons and subsequently amplifying downstream ISGs expression through a positive feedback loop (Fig. 4B red line) (49). Accordingly, we examined transcription levels of *IRF3* and *IRF7* and found that *IRF7* expression was significantly upregulated in the samples exposed to HuB20 and I226R compared with SY18, particularly in the I226R-exposed cells at 48 hpi (Fig. 4C). On the contrary, *IRF3* expression was similar among all the samples during infection. This phenomenon can also be found in infected cells (Fig. S4B). The alterations in *IRF3* and *IRF7* expression were further confirmed by qRT-PCR (Fig. 4E). We also experimentally validated the elevated expressions of *IFI6* and *IFIT1*, both of which are downstream members of IRF7, in HuB20- and I226R-exposed cells compared with SY18-exposed cells (Fig. 4E).

Furthermore, we detected a positive correlation between the *IRF7* expression and ISG scores in all three ASFV strain-exposed cells, especially for the HuB20 and I226R strains, whereas no significant correlation was found between the *IRF3* expression and ISG score (Fig. 4D). Collectively, our findings suggested that the IRF7-mediated interferon positive feedback loop pathway plays an important role in promoting ISGs transcription in attenuated and low virulent ASFV strain exposed host cells but not in high virulent strain exposed cells.

### Subclustering of PAMs revealed specific populations exhibiting varying responses to different virulent strains of ASFV

**Fig 5:**
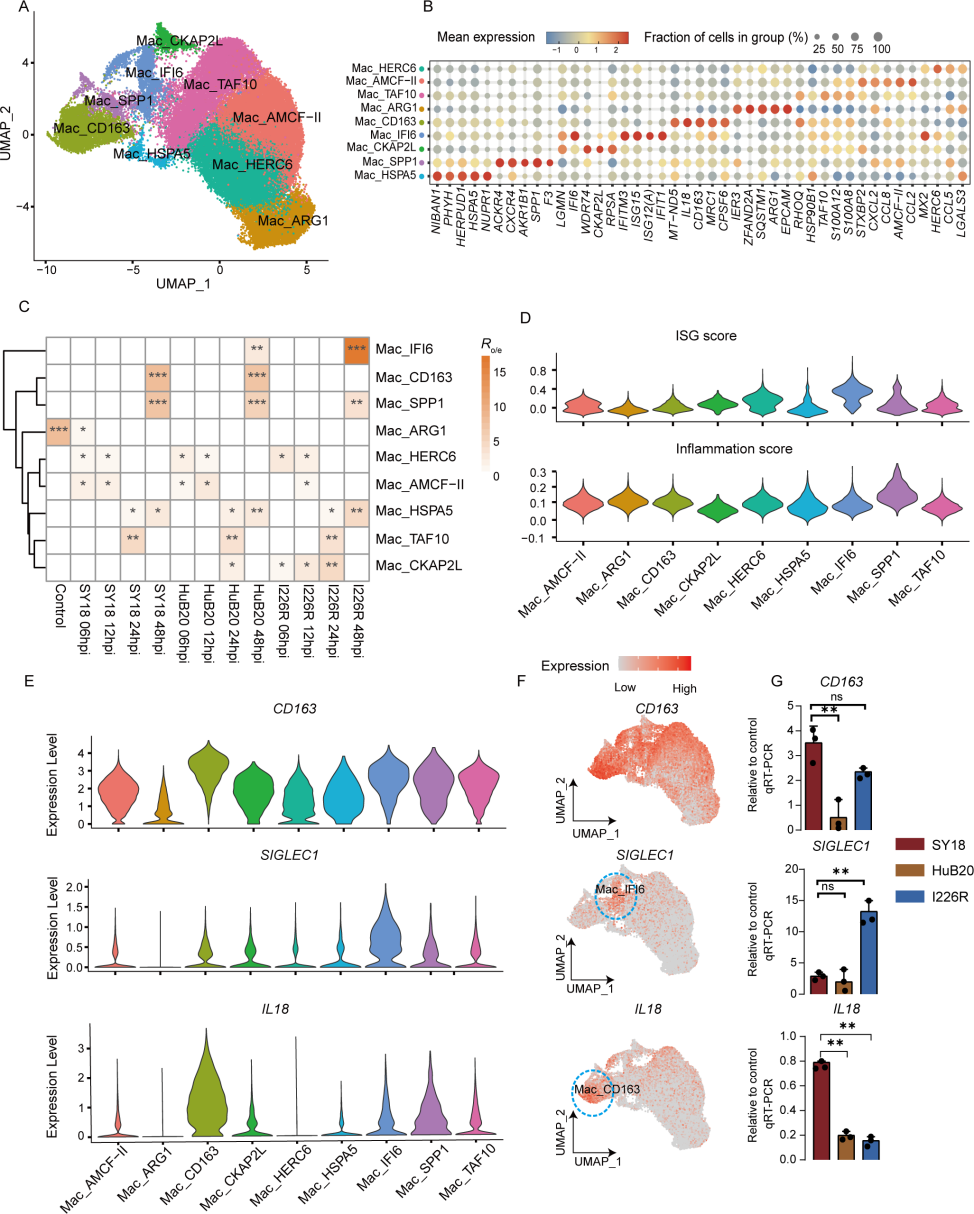
Subclustering of PAMs revealed specific populations that regulate host response to different virulent ASFV strains. (**A**) UMAP plots showing nine populations of PAMs among unexposed and exposed cells at each hpi. (**B**) Dot plot showing expression patterns of selected genes across indicated PAMs populations. (**C**) Heatmap showing the sample preference of each PAMs population estimated by *R*_o/e_. *R_O/e_>1, ** *R*_O/e_ > 5 and *** *R*_O/e_ > 10. (**D**) Violin plots showing ISG and inflammatory score for each PAMs population. (**E**) Violin plots showing the expression levels of *SIGLEC1, CD163,* and *IL18* across different PAMs populations. (**F**) UMAP plot showing expression levels of representative genes and corresponding PAMs populations are indicated with a blue circle. (**G**) Bar plots illustrating the transcription levels of *CD163*, *SIGLEC1* and *IL18 i*n PAMs exposed to three ASFV strains at 48 hpi using qRT-PCR. ns, not significant; **P < 0.01 and ***P < 0.001 (two-tailed t test). Data are shown as mean value ± SD, *n* = 3.

To figure out the impact of heterogeneity within PAMs exposed to ASFV, we employed an unsupervised clustering method to classify unexposed and exposed PAMs into nine populations with unique gene signatures (Fig. 5A and B; Table S2). We first quantified the enrichment of major populations in all samples by calculating the ratio of observed to expected cell numbers (*R*_o/e_) (24) (Fig. 5C). Our results indicate that cell populations of Mac_HERC6 and Mac_AMCF-II were enriched in cells exposed to all three ASFV strains at the early stage of infection (06, 12 hpi). Mac_HERC6 and Mac_AMCF-II contained high expressions of chemokine-related genes, including *CCL5*, *CCL2*, and *CCL8*. This suggests that cells in the Mac_HERC6 and Mac_AMCF-II populations might recruit immune cells to the local infected site during the early stages of infection.

Mac_IFI6 was primarily enriched in I226R- and HuB20-exposed cells, particularly in I226R-exposed cells at 48 hpi (Fig. 5C). This macrophage population exhibited notably elevated ISG scores, characterized by upregulated ISG expression (Fig. 5D and B). Moreover, *B2M*, *IFI6*, and *ISG15* exhibited higher expression levels compared with other ISG-related genes in Mac_IFI6 (Fig. S5A), suggesting that these three ISGs may play a crucial role in the host defense against ASFV. Our independent qRT-PCR results confirmed that the expression of these three ISGs was higher in I226R- and HuB20-exposed cells than in SY18-exposed cells at 48 hpi (Fig. S5B). Thus, we reasoned that I226R may induce the differentiation of host PAM cells into Mac_ IFI6, which produces a large amount of ISGs to defend against ASFV virulence. Furthermore, we found a significant negative correlation between ISG score and glycolysis and gluconeogenesis in Mac_IFI6 (Fig. S5C). This result supports previous studies that interferon response and glycolytic pathway could inhibit each other (37, 50).

CD163 is a scavenger receptor, and previous studies have indicated that CD163, along with SIGLEC1, collaborates to enhance host cell susceptibility to ASFV (33). Interestingly, *SIGLEC1* exhibited a high expression in Mac_IFI6, whereas *CD163* was highly expressed in nearly all the populations except MAC_ARG1 (Fig. 5E and F). Because Mac_IFI6 was the main population for cells exposed to I226R at 48 hpi, we anticipated that I226R-exposed cells at 48 hpi would display elevated *SIGLEC1* expression levels. Our independent qRT-PCR results confirmed this expectation (Fig. 5G). Additionally, there was no significant difference in *CD163* expression between SY18 and I226R (Fig. 5G). Thus, we reason those macrophages with high IFN pathway stimulation tend to express more *SIGLEC1*, which in turn enhances cell susceptibility to ASFV. This hypothesis might partly explain why attenuated and low virulent ASFV exhibited higher viral load than high virulent ASFV.

IL18 is a pro-inflammatory cytokine, and its overproduction could cause an exaggerated inflammatory burden and lead to tissue injury (51). Previous work revealed substantial upregulation of IL18 expression in the blood of pigs that succumbed to ASFV infection (10). We found that *IL18* was highly expressed in Mac_CD163 and Mac_SPP1, especially in Mac_CD163 (Fig. 5E and F). Because Mac_CD163 was a major cell type in cells exposed to SY18 at 48 hpi, we anticipated that SY18-exposed cells at 48hpi would display elevated *IL18* expression levels. Our qRT-PCR and ELISA results confirmed this expectation (Fig. 5G; Fig. S5D). Our results suggest that the virulent ASFV strain may induce the differentiation of host PAMs cells into Mac_CD163, which in turn produces a large amount of IL18 to increase the tissue damage and inflammatory burden of the host. This factor may contribute to the death of the host. Interestingly, we also found the activation of metabolism by cytochrome P450 was positively correlated with *IL18* expression in Mac_CD163 (Fig. S5E). Previous studies have shown that metabolites of cytochrome P450 (CYP450) could influence inflammatory response (52, 53). Our ELISA results demonstrated that the protein level of CYP2E1, one of the key subunits of CYP450, was higher in cells exposed to SY18 than those exposed to I226R (Fig. S5F). However, the role of metabolites of cytochrome P450 in controlling the inflammatory response of ASFV-exposed cells needs further investigation.

In summary, our analysis revealed diverse populations of PAMs induced temporally after infection with different virulent strains of ASFV. Specifically, we found that Mac_IFI6 exhibited the highest ISG expression and produced elevated levels of *B2M*, *IFI6*, and *ISG15*. Additionally, the Mac_CD163 population exhibited more pro-inflammatory cytokine *IL18,* which may increase the tissue damage and inflammatory burden of the host cells.

### CEBPB might be a key regulator of host response to ASFV infection

**Fig 6:**
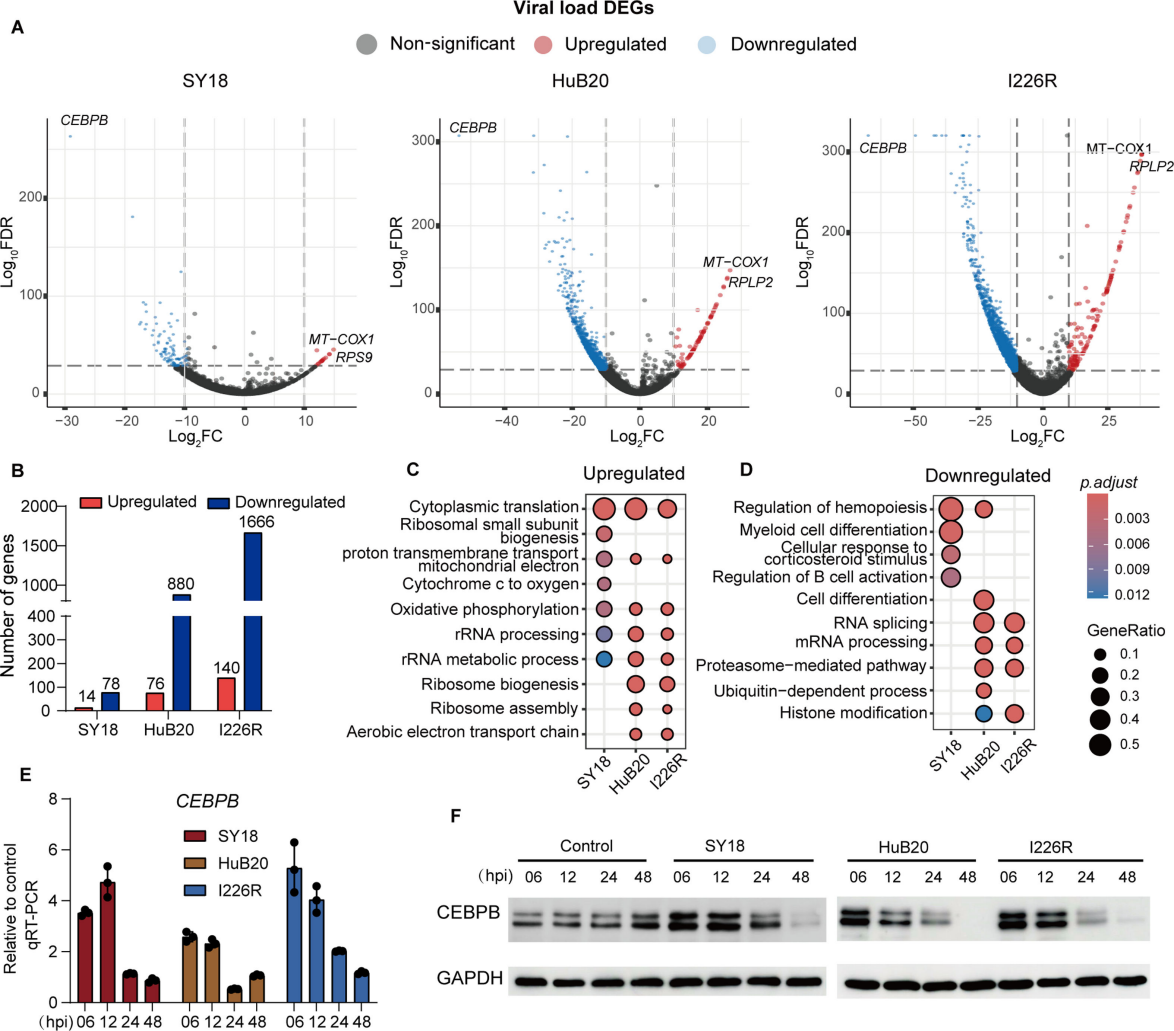
Association analysis between viral load and host genes identifies CEBPB as a target gene negatively regulated by ASFV replication. (**A**) Volcano plot showing the association between host gene expression and viral load within three ASFV strains exposed PAMs. The downregulated gene number refers to the number of host genes that were significantly negatively correlated with viral load, whereas the upregulated gene number refers to the number of host genes that were significantly positively correlated with viral load. (**B**) Boxplot showing the number of genes upregulated (positively correlated with viral load) or downregulated (negatively correlated with viral load) in PAMs exposed to three ASFV strains. (**C**) Dot plots showing GO terms of upregulated genes responded to increasing viral load in SY18, HuB20, and I226R. (**D**) Dot plots showing GO terms of downregulated genes responded to increasing viral load in SY18, HuB20, and I226R. (**E**) Bar plots showing the qRT-PCR results of relative transcriptional level of *CEBPB* in cells exposed to different ASFV strains at 6, 12, 24, and 48hpi. Data are shown as mean values ± SD, *n* = 3. (**F**) Western blot experiment showing results of CEBPB protein level in cells were unexposed or exposed to ASFV SY18, HuB20, and I226R, respectively (MOI = 3), at 6, 12, 24, and 48 hpi.

Next, in cells exposed to different ASFV strains, we looked for associations between viral load and host gene transcript levels to identify genes regulated by viral gene expression (22) (Fig. 6A; Table S2). To our surprise, we observed that the number of host genes showing altered expression was the highest in cells exposed to I226R, followed by HuB20, whereas the SY18 exposed cells had the least host genes with an altered expression, which could be attributed to the superior immune escape functions of SY18 (12, 46) (Fig. 6B). Moreover, we observed that a higher number of host genes exhibited decreased expression in response to viral gene expression compared with those showing increased expression (Fig. 6B). This result, in conjunction with our previous findings that the replication of ASFV may affect the diversity of host gene expression (Fig. S2B), indicates that ASFV replication within the host cells not only suppresses the expression levels of host genes but also inhibits the diversity of host gene expression.

Consistently among all three ASFV strains, several mitochondrial genes, including *MT-COX1*, *MT-COX2*, and *MT-COX3*, were upregulated as viral load increased. This is consistent with our findings of activated host metabolic pathways post-ASFV infection (Fig. 3B). Furthermore, we found ribosomal genes, including *RPS9* and *RPLP2*, were upregulated as viral load increased. GO analysis showed that the upregulated genes were enriched in the ribosome and mitochondrial related pathways (Fig. 6C). These results indicated that ASFV could enhance host cell metabolism and facilitate the formation of ribosomes to supply energy and proteins necessary for viral replication.

In contrast, the downregulated genes were enriched in mRNA processing and proteasome-mediated pathways, suggesting ASFV could negatively regulate the host transcription and protein stability (Fig. 6D). Interestingly, in cells exposed to all three strains of ASFV, the transcription factor *CEBPB* showed a significant negative correlation with viral load (Fig. 6A), indicating that viral replication could potentially impede *CEBPB* expression. We confirmed this result by conducting independent qRT-PCR and western blot analysis to detect the transcriptional and protein levels of CEBPB (Fig. 6E and F). The results indicated that both mRNA and protein levels of CEBPB in host cells decreased over time with prolonged ASFV infection, and this reduction was observed in cells exposed to all three ASFV strains. CEBPB belongs to the CCAAT/enhancer binding protein family and is closely associated with inflammation observed in various viral and traumatic diseases (54). Previous studies have reported a correlation between the expression of CEBPB and specific inflammatory factors, such as *IL17*, which have demonstrated inhibitory effects on viral replication (55). Therefore, we speculated that CEBPB might be a key regulator to host response upon ASFV infection, and thus, activation of CEBPB might be beneficial to the host defense to ASFV. Further studies will be needed to fully discriminate the function of CEBPB in ASFV replication.

## DISCUSSION

**Fig 7:**
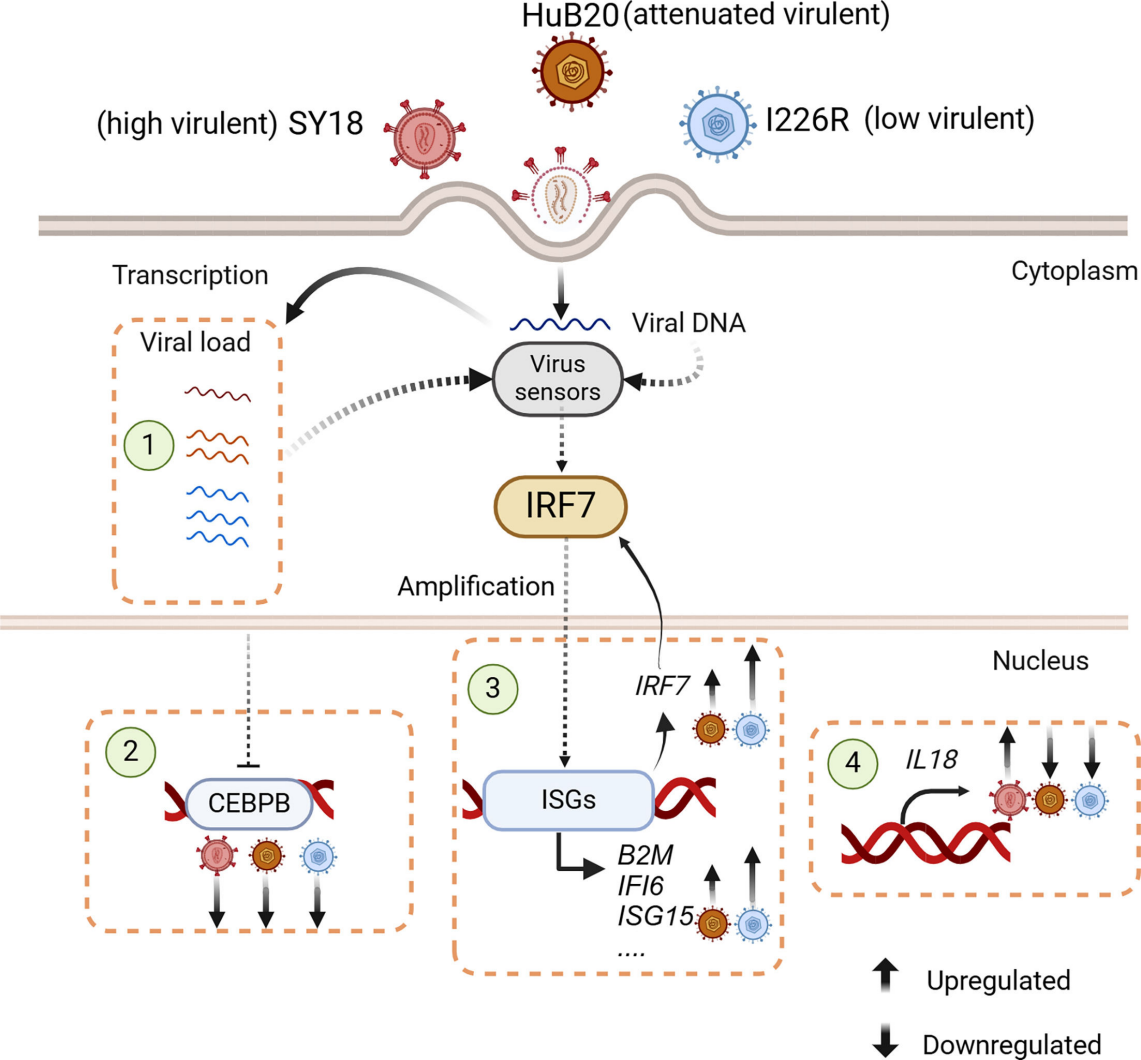
Graphical summary of the differences in PAM cells exposed to ASFV strains of varying virulence. Schematic overview of the work: (1) attenuated and low virulent ASFV strains, HuB20 and I226R, exhibited higher viral load compared with the highly virulent strain SY18; (2) CEBPB was identified as a target gene negatively regulated by ASFV viral load in all three AFSV strains; (3) IRF7-mediated interferon positive feedback loop pathway contributed to the promotion of ISGs transcription in cells exposed to HuB20 and I226R, but not SY18; and (4) pro-inflammatory cytokine IL18 was highly expressed in cells exposed to SY18 compared to cells exposed to HuB20 and I226R at 48 hpi.

ASF is characterized by hemorrhage and high mortality, resulting in huge economic losses in the global pig industry. However, the underlying mechanisms of ASFV virulence and pathogenicity remain unclear. Here, we present a comprehensive single-cell mRNA profiling of PAMs exposed to three distinct virulent ASFV strains: SY18, HuB20, and I22R *in vitro*. These ASFV strains exhibit varying degrees of virulence, ranging from high to low. We found that attenuated and low-virulence ASFV strains have higher viral loads than highly virulent strains, which may result from upregulated RNA polymerase subunit genes expression. Moreover, our study highlights IRF7 mediated positive feedback loop to activation of the interferon signaling pathway in cells exposed to attenuated and low virulent ASFV strains. Our investigation also unveiled that some PAM populations, such as Mac_IFI6 and Mac_CD163, could produce a higher level of ISGs or *IL18* to regulate the host response to different virulent ASFV strains. Finally, we found *CEBPB* as a target gene negatively regulated by ASFV replication in all three AFSV strains (Fig. 7).

We discovered higher viral load in cells exposed to attenuated and low virulent ASFV strains compared with cells exposed to high virulent strains. On the other hand, our results also indicated that I226R viral titer is slightly lower than that of SY18, although the transcript abundance of I226R was higher than that of SY18. This imply that viral transcripts from I226R could not be efficiently translated and assembled into new virions. We speculate that host cell immune responses play a crucial role in this phenomenon. For instance, it has been reported that the *OAS1* gene, one of a ISGs, enhances the proteasomal degradation of the ASFV P72 protein by promoting TRIM21-mediated ubiquitination (56). The ASFV P72 is the major capsid protein of ASFV (27), and its degradation could impact the synthesis of ASFV virions. In our single-cell data set, the expression of *OAS1* in host cells exposed to HuB20 and I226R was significantly higher than that in cells exposed to SY18 (data not shown). This observation suggests *OAS1* may mediate the degradation of the ASFV P72 protein in I226R-exposed cells. However, the detailed mechanism needs further study.

*Zheng* and *Zhu* conducted single-cell sequencing of PAM cells and spleens exposed to ASFV (7, 25). In terms of ASFV gene expression, they found that the *MGF_110–7L, MGF_110–5L-6L, MGF_110–3L,* and *MGF_110–4L* genes were the top-ranked viral genes during infection, which is consistent with our data. However, the above article only infected the host with only one strain of ASFV, and there was no comparison between different ASFV strains. In our data, we observed varying expression levels of specific ASFV genes across different ASFV strains. For example, *CP2475L* and *CP530R* exhibited higher expression levels in low virulent strains than in high virulent strain, whereas *I226R* and *L11L* demonstrate increased expression in high virulent strain.

Interestingly, we also found that a low-virulent ASFV strain, I226R, may upregulate the expression of viral RNA polymerase subunit genes, resulting in accelerated transcription rate. Genomic sequencing data for the I226R strain indicate that in addition to the knockout of the I226R gene, three additional mutations are present. Two of these mutations did not alter the ORFs or peptide lengths: one is a 7-base insertion in the intergenic region between *E423R* and *E301R*, and the other is a single-base mutation in the *E199L* ORF resulting in an amino acid change from Glu to Val. The last one in the *MGF_360–11L* ORF, the absence of base C at position 26716 of the I226R genome led to the truncation of the peptide from 353 to 89 amino acids. Previous research has suggested that the *I226R* gene of ASFV has multiple functions in suppressing host innate immunity expression (12). E199L can mediate ASFV entry by enabling membrane fusion and core penetration and promotes cell autophagy (57, 58). MGF_360–11L can negatively regulate cGAS-STING-mediated inhibition of type I interferon production (59). However, to date, there have been no studies reporting whether the I226R gene or these three mutations are associated with the viral RNA polymerase subunit genes or viral transcription rate of ASFV. This area remains worthy of further investigation.

Additionally, it is worth mentioning that readthrough transcription events, which occur frequently during ASFV transcription, can disrupt the expression of downstream genes (60). Readthrough transcription in ASFV complicates the accurate estimation of mRNA using next-generation sequencing (NGS) technology. This limitation arises from the fact that NGS methods often generate short read lengths, making it challenging to distinguish between genuine transcription events and readthrough transcripts. Future studies are needed to gain a more accurate transcriptional profile of different ASFV strains following infection by employing third-generation sequencing (TGS) technology.

In terms of host gene expression, Zheng et al. and Zhu et al. found that PAMs exposed to ASFV activated interferon and inflammatory responses, consistent with our previous findings (7, 25). In addition, we also found that PAMs exposed to low virulent strains activated the immune response, while cells exposed to high virulent strain suppressed the immune response. To facilitate viral growth, ASFV could transcribe some genes to inhibit the activation of host cell antiviral pathways, especially for interferon pathways (59, 61). IRF3 and IRF7 are important transcription factors in interferon pathways. Previous studies have mainly focused on elucidating some ASFV proteins that could regulate the modification and stability of IRF3 to regulate the immune response (45, 61). An interesting finding in this study highlights the role of IRF7-mediated positive feedback loop in activating the interferon signaling pathway in cells exposed to HuB20 and I226R ASFV strains. It has been reported that IRF7 has a major role in feedback amplification of the interferon signaling pathway upon the synthesis of new IRF7 molecule (62). So far, only a few studies have reported the relationship between ASFV proteins and IRF7 (59, 63), further investigation is needed to substantiate our speculation.

Consequently, we used an unsupervised clustering method to classify PAMs into nine populations. We found that ISGs had high expression levels in Mac_IFI6 which were mainly distributed in low virulent ASFV strain exposed cells. Additionally, we observed that *B2M*, *IFI6*, and *ISG15* were the three most highly expressed ISGs in Mac_IFI6. B2M is a crucial subunit of the major histocompatibility complex (MHC) class I and plays an important role in antigen presentation (64). However, little is known about how B2M is utilized by PAMs to present antigens from ASFV and how it interacts with other immune cell types in response to ASFV antigens. IFI6 and ISG15 have been reported to possess antiviral properties against several viruses, including Zika virus (65) and hepatitis C virus (66), but few studies have clarified their role in ASFV. Therefore, more research should be done to elucidate the mechanisms of B2M, IFI6, and ISG15 in ASFV-exposed cells in the future.

We observed that Mac_CD163 is primarily distributed in the high virulent ASFV strain exposed cells. Mac_CD163 exhibited high expression of *IL18*, a pro-inflammatory cytokine. Overproduction of IL18 can result in an exaggerated inflammatory burden and tissue injury (51). Previous reports indicate the expression of IL18 was significantly elevated after the pig was exposed to high virulent ASFV and remained until the host died (10). These results imply that IL18 may have an important role in the ASFV virulence. Additionally, we found a mild positive correlation between *IL18* expression and the activity of xenobiotics processed by cytochrome P450 in Mac_CD163. Previous research has demonstrated that metabolites of xenobiotics processed by cytochrome P450 can influence the inflammatory response (52, 53). In our future experiments, we intend to investigate the relationship between cytochrome P450 and IL18 in Mac_CD163 during ASFV infection.

Energy and ribosomes are needed for ASFV to construct the virus factory to facilitate viral protein translation and virus assembly (67). Our results indicated the activation of the TCA pathway following ASFV infection which support previous report that ASFV infection promotes the TCA cycle (37). Besides, we found that many ribosome-related genes, such as *RPS9* and *RPLP2*, were upregulated during ASFV infection. Further investigation is warranted to elucidate the influence of interfering with these metabolic pathways and ribosome-related genes on ASFV.

We acknowledge three limitations in our study. Firstly, it is worth noting that our study involved the utilization of three distinct ASFV strains. However, considering the vast range of ASFV diversity, this number could be considered relatively small. The results should be validated in another prospective study. Second, due to the limitations of experimental conditions, our cells were collected from *in vitro* experiments, and the results we obtained should be further elucidated by *in vivo* experiments. Third, our focus was exclusively on analyzing the impact of ASFV on PAMs, neglecting the examination of interactions among different cell types that could potentially play a crucial role in ASFV infection. Further analysis of interactions among cell types after ASFV infections is needed.

### Conclusions

In summary, our work establishes a comprehensive transcriptome profile of virus and host dynamics in different virulent ASFV-exposed PAMs at the single-cell level. We revealed attenuated and low-virulence ASFV strains have higher viral loads than highly virulent strains, which may result from upregulated RNA polymerase subunit genes expression. Furthermore, our study highlights IRF7 mediated positive feedback loop to activation of the interferon signaling pathway in cells exposed to HuB20 and I226R ASFV strains. Moreover, in cells exposed to high virulent ASFV, our investigation revealed some specific PAMs populations, such as Mac_IFI6 and Mac_CD163, which could produce higher levels of ISGs or *IL18* to regulate host response to different virulent ASFV strains. This study enhances our understanding of virus–host interactions and the molecular events involved in viral replication and pathogenesis.

## Data Availability

Single-cell RNA sequencing data are publicly accessible through the link https://ngdc.cncb.ac.cn/gsa/browse/CRA022265. This paper does not report original code. The R scripts used for calculating viral load are on GitHub (https://github.com/zhaoxiaoyan9225/ASFV-scRNA), and other R scripts used for analysis and visualization are available upon reasonable request to the corresponding author.

## SUPPLEMENTAL MATERIAL

Fig. S1 jvi.02035-24-s0001.tif Landscape of single-cell transcriptome dynamics in PAMs exposed to ASFV.

Fig. S2 jvi.02035-24-s0002.tif Attenuated and low-virulence ASFV strains tend to exhibit higher viral load compared to highly virulent ASFV strain.

Fig. S3 jvi.02035-24-s0003.tif Metabolic state and pseudo-time trajectory of PAMs exposed to different ASFV strains.

Fig. S4 jvi.02035-24-s0004.tif Interferon pathways were activated through the IRF7-mediated positive feedback loop in attenuated and low virulent ASFV exposed PAMs.

Fig. S5 jvi.02035-24-s0005.tif Subclustering of PAMs revealed specific populations that regulate host response to different virulent ASFV strains.

Supplemental legends jvi.02035-24-s0006.docx Legends for Fig. S1 to S5.

Supplemental text jvi.02035-24-s0007.txt Genome sequencing data of I226R.

Table S1 jvi.02035-24-s0008.xlsx Information related to Fig. 1 to 3 and Fig. S3.

Table S2 jvi.02035-24-s0009.xlsx Information related to Fig. 4 to 6 and Fig. S4 and S5.

Table S3 jvi.02035-24-s0010.xlsx Information related to methods.

ASM does not own the copyrights to Supplemental Material that may be linked to, or accessed through, an article. The authors have granted ASM a non-exclusive, world-wide license to publish the Supplemental Material files. Please contact the corresponding author directly for reuse.
