# Supplementary figures and images for: Diversities of African swine fever virus host-virus dynamics revealed by single-cell profiling
Source: J Virol. 2025 Feb 11;99(3):e02035-24. doi: 10.1128/jvi.02035-24 (PMC11917525; doi:10.1128/jvi.02035-24)

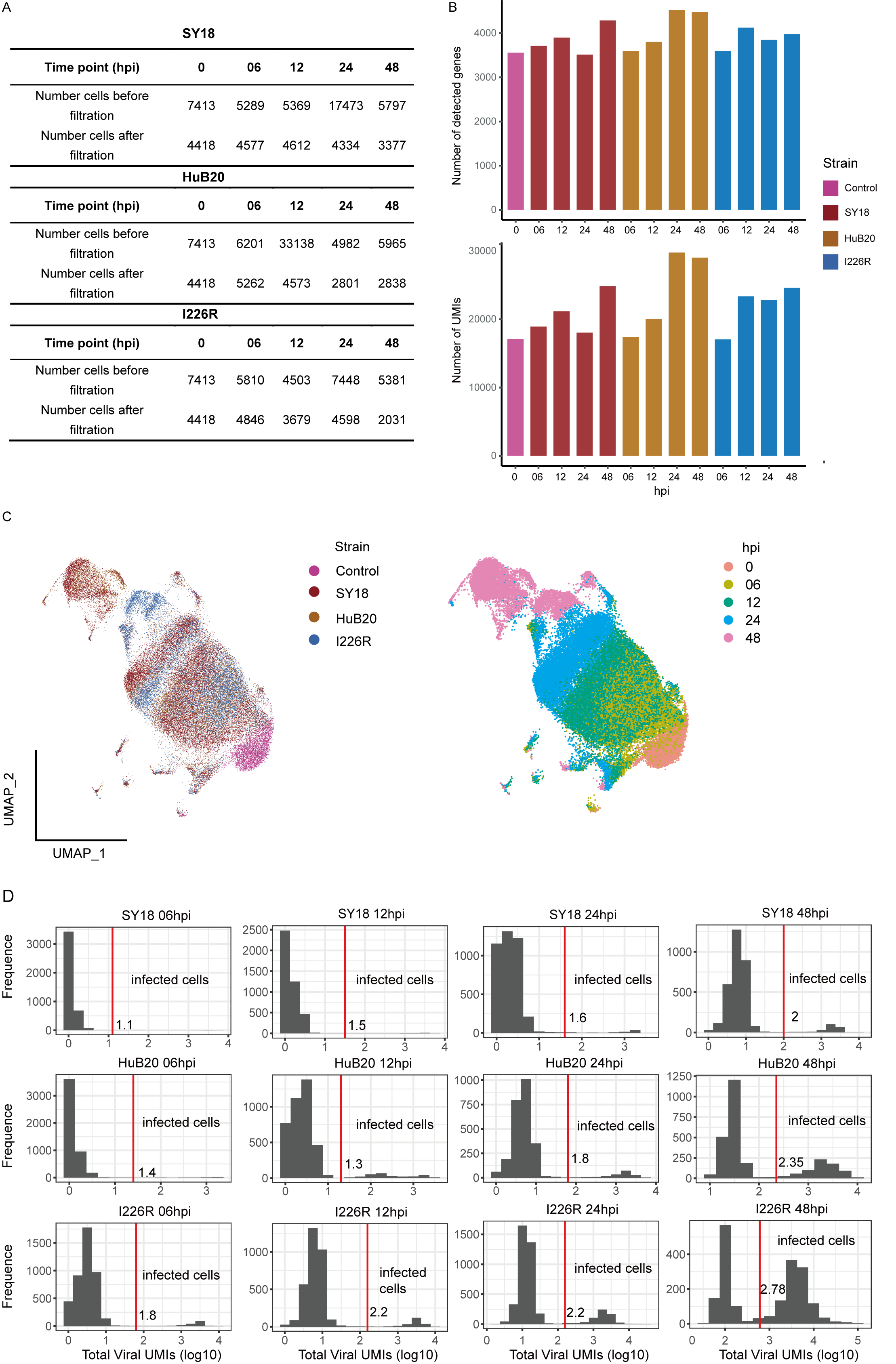

Supplement: Fig. S1 — Landscape of single-cell transcriptome dynamics in PAMs exposed to ASFV. [file jvi.02035-24-s0001.tif]

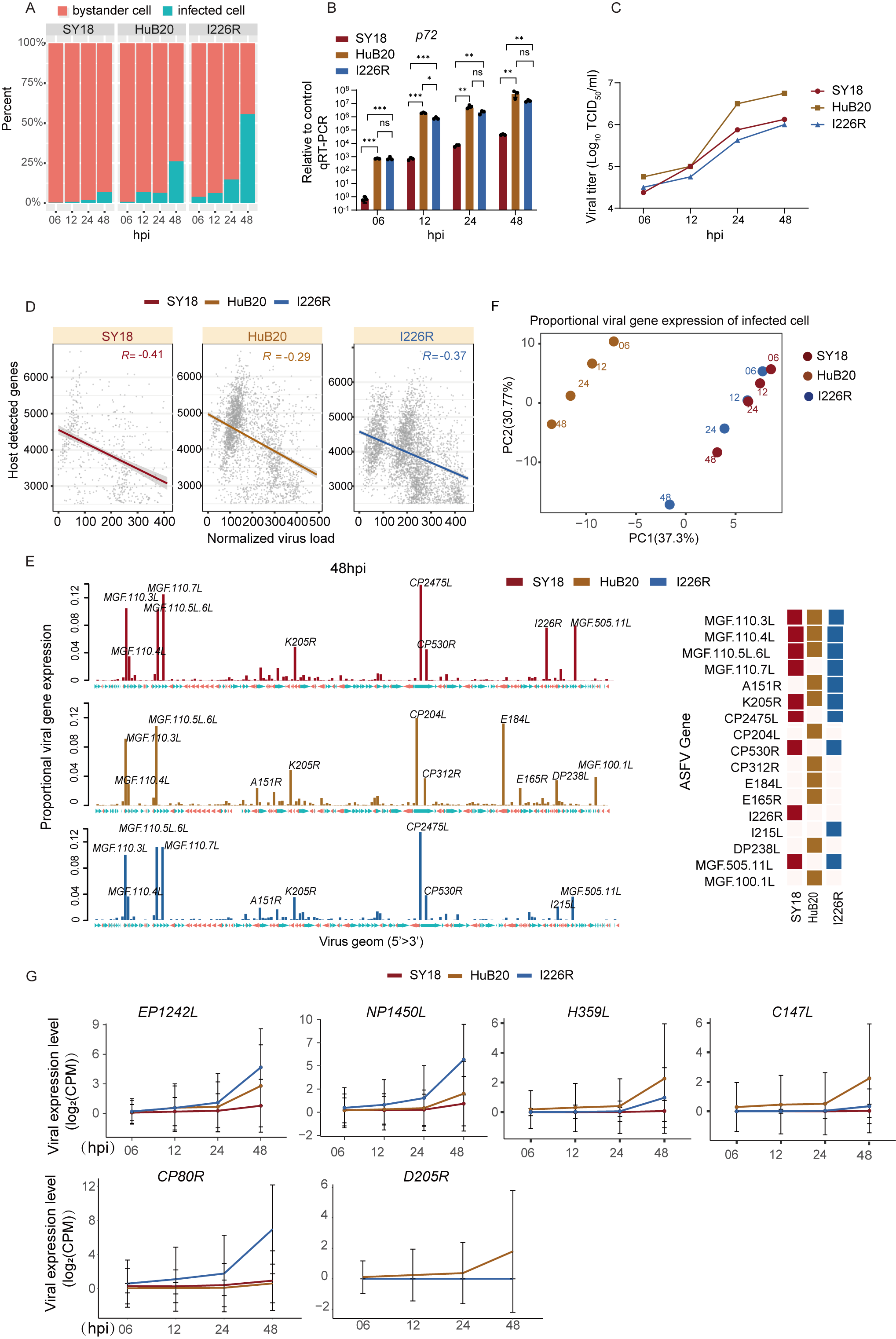

Supplement: Fig. S2 — Attenuated and low-virulence ASFV strains tend to exhibit higher viral load compared to highly virulent ASFV strain. [file jvi.02035-24-s0002.tif]

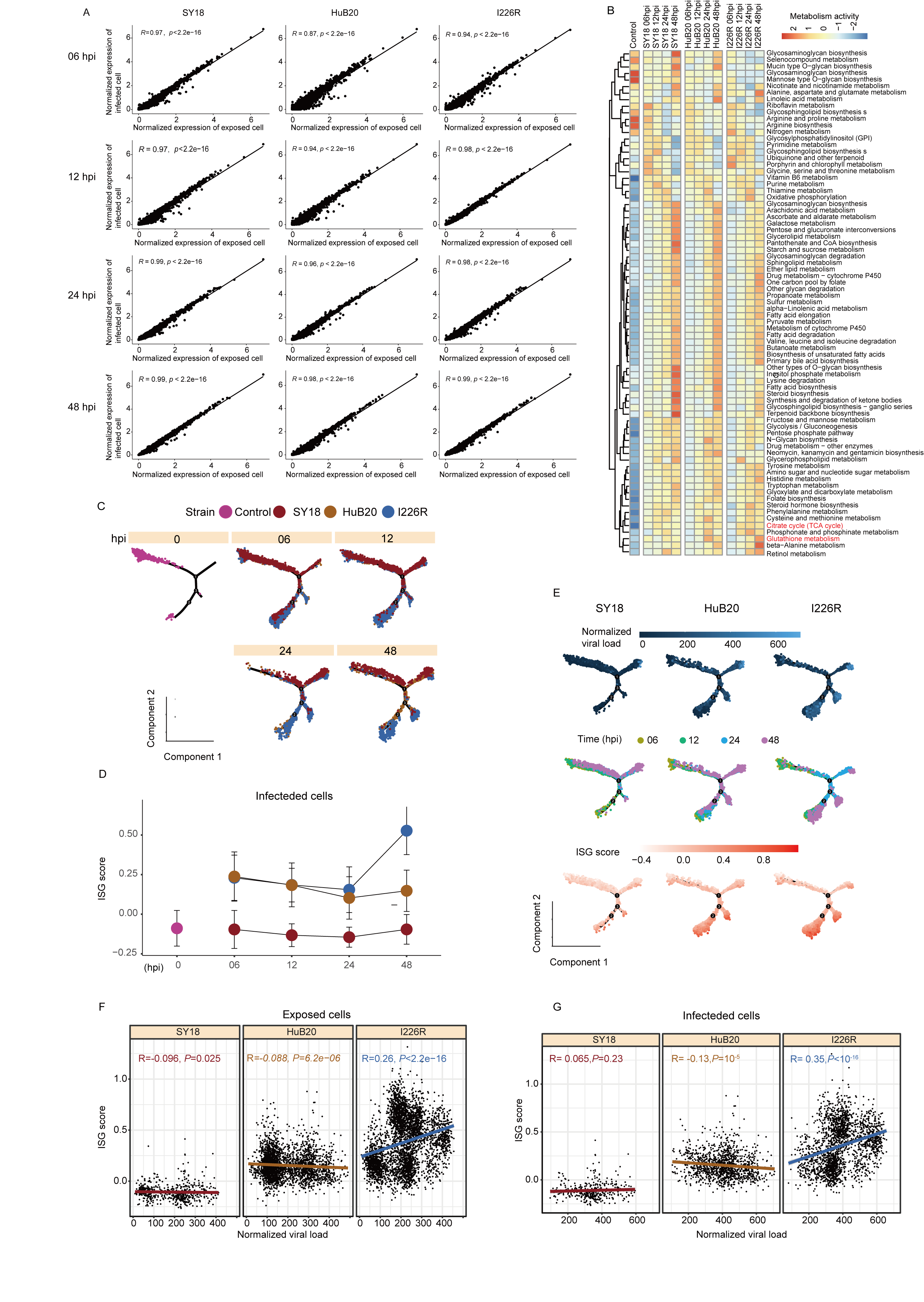

Supplement: Fig. S3 — Metabolic state and pseudo-time trajectory of PAMs exposed to different ASFV strains. [file jvi.02035-24-s0003.tif]

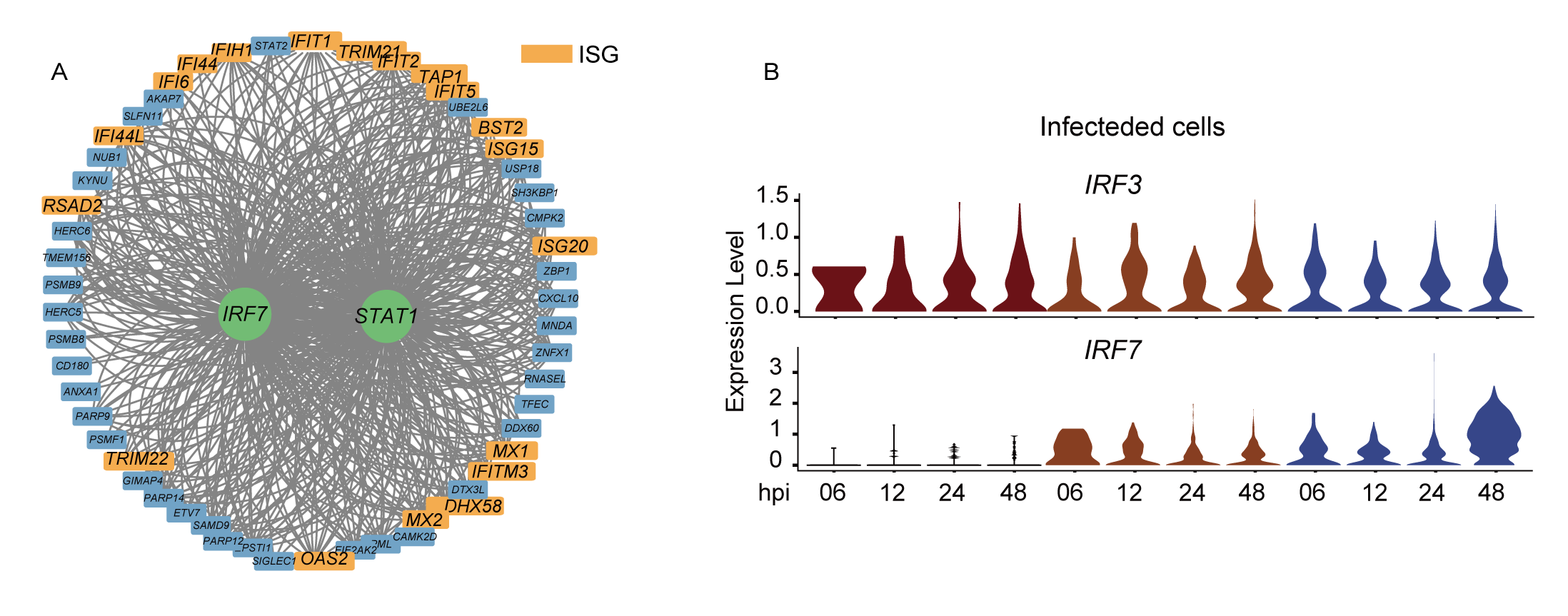

Supplement: Fig. S4 — Interferon pathways were activated through the IRF7-mediated positive feedback loop in attenuated and low virulent ASFV exposed PAMs. [file jvi.02035-24-s0004.tif]

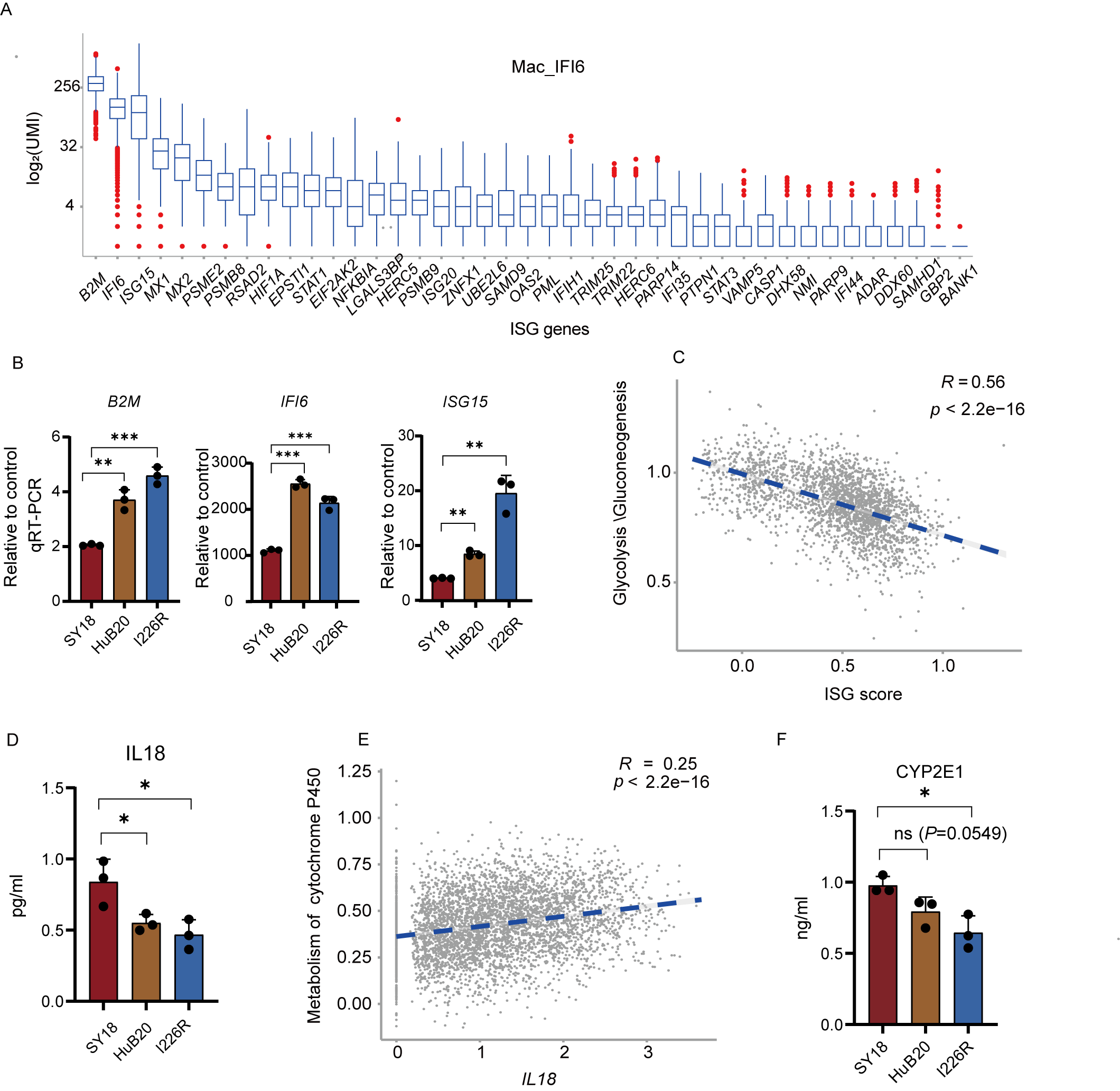

Supplement: Fig. S5 — Subclustering of PAMs revealed specific populations that regulate host response to different virulent ASFV strains. [file jvi.02035-24-s0005.tif]
