# Supplementary material for: Diversities of African swine fever virus host-virus dynamics revealed by single-cell profiling
Source: J Virol. 2025 Feb 11;99(3):e02035-24. doi: 10.1128/jvi.02035-24 (PMC11917525; doi:10.1128/jvi.02035-24)
Supplement: Supplemental legends — Legends for Fig. S1 to S5. [file jvi.02035-24-s0006.docx]

**Supplementary Figure Legends**

**Supplementary Figure 1. Landscape of single-cell transcriptome dynamics in PAMs exposed to ASFV.**

1. Table showing the number of cells exposed to different ASFV strains. The number of cells before and after filtration is indicated.
2. Bar plots showing the number of detected host genes (upper) and UMIs (down) in cells from different samples.
3. UMAP plots showing the distribution of cells exposed to different strains and hpi.
4. Distribution of ASFV UMIs in all collected cells across each sample. Red line indicates thresholding of infected cells that calculated by OTSU algorithm. Cells with more ASFV UMIs than threshold were attributed to infected cells. Cells with fewer ASFV UMIs than threshold were attributed to bystander cells

**Supplementary Figure 2. Attenuated and low-virulence ASFV strains tend to exhibit higher viral load compared to highly virulent ASFV strain.**

1. Bar plots showing the percentage of bystander cell and infected cell in each sample.
2. Bar plots showing viral gene *p72* transcription by qRT-PCR. ns, not significant; ***p* < 0.01 and ****p* < 0.001 (two-tailed t test). Data are shown as mean value ± SD, n=3.
3. Line plot showing viral titer of three ASFV strains at 6,12,24,48 hpi.
4. Scatter plot showing the correlation of number of detected host genes (vertical axis) with normalized virus load (horizontal).
5. Left panel: bar plots showing the proportional viral gene expression in exposed cells at 48hpi (order by viral gene location in the genome). Right panel: heatmap showing highly proportional expressed viral genes (proportional expression >0.02) in each ASFV strain exposed cells at 48hpi.
6. PCA plot showing the distribution of each sample by the proportional viral gene expression in infected cell.
7. Line plots showing the expression levels of representative viral genes. Gene expression levels are quantified with counts per million mapped reads (CPM). Data are shown as mean value ± SEM.

**Supplementary Figure 3. The metabolic state and pseudo-time trajectory of PAMs exposed to different ASFV strains.**

(A) Scatter plot showing the correlation of host gene expression between exposed and infected cells in each sample.

(B) The heatmap showing the activities of multiple metabolic pathways in each sample.

(C) The pseudo-time trajectory reconstructed by Monocle. Cells are colored based on ASFV strains.

(D) Line plot showing ISG score for the infected cells at each hpi. The lines indicate the median trend of the ISG score.

(E) The pseudo-time trajectory reconstructed by Monocle. Cells are colored based on viral load, hpi and ISG score.

(F) Scatter plots showing the relationship between ISG score and normalized viral load in exposed cells. Spearman correlation coefficients (*R*) and correlation test *p* value are indicated.

1. Scatter plots showing the relationship between ISG score and normalized viral load in infected cells. Spearman correlation coefficients (*R*) and correlation test *p* value are indicated.

**Supplementary Figure 4. The interferon pathways were activated through the IRF7-mediated positive feedback loop in attenuated and low virulent ASFV exposed PAMs**

1. Gene regulation network showing potential key regulators *IRF7* and *STAT1* and their target genes. The orange color indicates the ISG-related genes and the green indicates the transcription factors.
2. The violin plots show the expression levels of *IRF3* and *IRF7* in infected cells.

**Supplementary Figure 5. Subclustering of PAMs revealed specific populations that regulate host response to different virulent ASFV strains**.

1. Box plots showing the expression levels of ISGs in Mac_IFI6. The red dots are outliers.
2. Bar plots showing the qRT-PCR results of the relative transcriptional level of *B2M, IFI6* and *ISG15* in cells exposed to different ASFV strains at 48hpi. ns, not significant; ***p* < 0.01 and ****p* < 0.001 (two-tailed t test). Data are shown as mean value ± SD, n=3.
3. Scatter plots showing the relationship between ISG score and metabolism activity of Glycolysis\Gluconeogenesis in Mac_IFI6 strain. Spearman correlation coefficients (*R*) and correlation test *p* value are indicated.
4. Bar plots showing ELISA results of the IL18 protein level in cells exposed to different ASFV strain at 48hpi. ns, not significant; ns, not significant; ***p* < 0.01 and ****p* < 0.001 (two-tailed t test). Data are shown as mean value ± SD, n=3.
5. Scatter plots showing the relationship between the expression level of *IL18* and the metabolism activity of cytochrome P450 in Mac_CD163 population. Spearman correlation coefficients (*R*) and correlation test *p* value are indicated.
6. Bar plots showing ELISA results of the CYP2E1 protein level in cells exposed to different ASFV strain at 48hpi. ns, not significant; ** *p* < 0.01 and *** *p* < 0.001 (two-tailed t test). Data are shown as mean value ± SD, n=3
